# Synthetic strategies for the fluorescent labeling of epichlorohydrin-branched cyclodextrin polymers

**DOI:** 10.3762/bjoc.10.319

**Published:** 2014-12-16

**Authors:** Milo Malanga, Mihály Bálint, István Puskás, Kata Tuza, Tamás Sohajda, László Jicsinszky, Lajos Szente, Éva Fenyvesi

**Affiliations:** 1 CycloLab Cyclodextrin Research and Development Laboratory Ltd, Illatos 7, Budapest, H-1097 Hungary

**Keywords:** coumarin, fluorescein, functionalized monomers and polymers, nitrobenzofurazan, rhodamine

## Abstract

The fluorescent tagging of cyclodextrin derivatives enlarges their spectroscopic properties thus generating chemosensors, biological tools for visualization and sophisticated photoresponsive devices. Cyclodextrin polymers, due to the cooperative interactions, exhibit additional properties compared to their monomeric counterpart. These macromolecules can be prepared either in well water-soluble form or as gels of high swelling. Two versatile synthetic strategies for introducing a fluorescent tag (rhodamine, fluorescein, nitrobenzofuran or coumarin) into the water-soluble epichlorohydrin branched cyclodextrin polymers were worked out and compared. The fluorescent labeling was realized in three steps: 1) building in azido moieties, 2) transforming the azido groups into amino groups and 3) coupling the proper fluorescent compound to the amino groups. The other strategy started by functionalization of the monomer prior to the branching. Either the fluorescent-labeled monomer or the intermediate azido derivative of the monomer was branched. Further tuning of the properties of the polymer was achieved via branching of the methylated cyclodextrin derivative. The key intermediates and the fluorescent final products were characterized by various spectroscopic techniques and capillary electrophoresis. The applied synthetic routes were evaluated based on the molecular weight, cyclodextrin content of the products and the efficiency of labeling.

## Introduction

Cyclodextrins (CDs) are cyclic oligosaccharides consisting of 6, 7 or 8 glucopyranose units (α-, β- or γ-CD, respectively). The capability for complexation, as well as their ability to stabilize and solubilize guest compounds, makes these substances prime candidates for incorporation in delivery systems for valuable and sensitive materials [1–3]. However, CDs, in particular the most common β-CD, have some limitations both in molecular dimensions and physicochemical properties like water solubility. The low water solubility can present a problem when there is a dedicated use of CDs in a delivery system that has contact with water, or when CD is to be used for the removal of specific component(s) of a water-based process. In addition, free CDs are more difficult to recover for commercial recycling.

A solution to these challenges is encoded in polymeric cyclodextrin structures. The cyclodextrins fixed into polymeric structures behave differently from their monomeric counterparts [4–6]. Depending on the requirements of application both highly soluble and water-insoluble polymers (gels, resins) can be produced. Furthermore, the polymeric structure can constrain the rate of rotation of the CDs. The close proximity of the adjacent cavities can enhance their complexation ability, while the high degree of branching may result in reduced complexation owing to the steric hindrance. These polymers containing CDs can also provide additional functionalities in that they can regulate the release of substances into water-based systems.

For the branching of cyclodextrin rings any bi- or polyfunctional reagents able to react with the hydroxy groups can be used. The most widely-used branching reagent for the production of branched CD polymers is epichlorohydrin [7–8]. The reaction conditions, the work-up, the process of purification and the characterization for the epichlorohydrin branched water soluble polymers obtained from native cyclodextrins are well established processes [9–10]. The characteristics of the cyclodextrin polymers can be modulated by appending neutral or ionic functional groups either by branching the proper cyclodextrin derivatives (pre-branching modification) or by derivatization of the cyclodextrin polymers (post-branching modification).

Several examples of both modification types of these macromolecules with additional groups are reported in the literature [5] and an update review has been recently published [11]. Cationic and anionic cyclodextrin polymers were synthesized by simultaneous modification and branching from β-CD, epichlorohydrin and choline chloride (for cationic polymer) or chloroacetic acid (for anionic polymer) in an one-step polycondensation process and used for layer-by-layer film formation [12]. The synthesis and characterization of a nanosized quaternary ammonium β-CD polymer with high load capacity of the anticancer drug doxorubicin has been described as well as its application as drug delivery carriers across the blood–brain barrier [13].

The introduction of methyl groups into the CD polymers generates structures with peculiar properties, such as enhanced solubility and solubilizing effect, although only a few examples are found in the literature [14–15]. The introduction of a fluorescent tag into a water-soluble epichlorohydrin-branched CD polymer leads to compounds with enhanced spectroscopic properties, but it has been rarely described. Zohrehvand and Evans reported the synthesis, characterization and fluorescence studies of a water-soluble 2-naphthol-containing β-CD-epichlorohydrin copolymer [16], but in this case the fluorophore is randomly introduced into the polymeric matrix.

Recently Mamba’s group [17] described an example of fluorescent tagging of epichlorohydrin branched β-CD polymer based on a pre-branching modification approach. In his work the β-CD was first modified with an azo dye making it less soluble in water. The modified β-CD was then copolymerized with epichlorohydrin in a ratio that resulted in the formation of a water-soluble copolymer. The azo dye modified β-CD epichlorohydrin copolymer was used as a molecular fluorescent sensor for the detection of chloroform, one of the chlorination byproducts in water.

Here, we report the controlled, regioselective fluorescent labeling of neutral and cationic epichlorohydrin branched β-CD polymer with four different dyes (rhodamine, fluorescein, coumarin and nitrobenzofurazan). Our goal was the development of versatile synthetic strategies, yielding key intermediates of wide applicability for the fluorescent labeling of CD polymers. The ad-hoc characterization of all the synthesized compounds was performed for comparative evaluation of these strategies.

## Results and Discussion

**Scheme 1:**
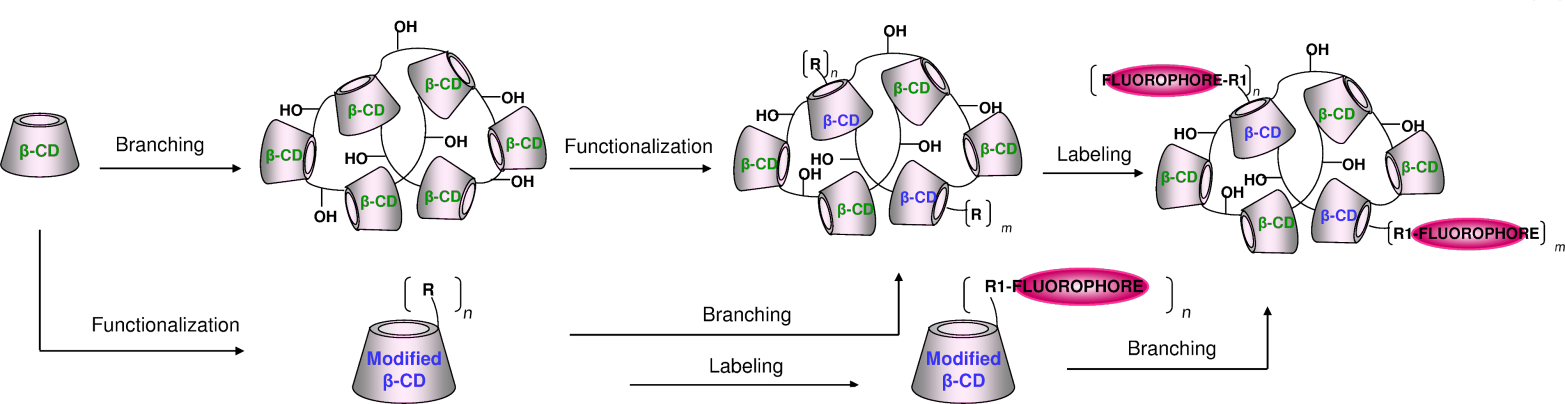
Schematic representation of the various synthetic routes for the introduction of an anchoring group (R) for the fluorescent dye into the CD scaffold and labeling prior to or after branching.

Two strategies were built-up for the fluorescent labeling of epichlorohydrin branched polymers and the approaches are based on the introduction of a dedicated anchoring group for the fluorophore (functionalization) into the CD scaffold. The general scheme for the two synthetic approaches is shown in Scheme 1.

The first strategy is based on the post-branching functionalization (Scheme 1, upper part). In this case, the introduction of the anchoring group for the fluorescent labeling is performed after the formation of the polymer. According to this procedure, the branching agent, epichlorohydrin, interacts with the maximum number of hydroxy groups available per CD ring, since the branching occurs between the unmodified cyclodextrin. As a consequence, the branching process is more efficient compared to the branching of the modified CD monomer thus leading to a macromolecule with higher molecular weight. A slight modification of this procedure consists in a preliminary modulation of the polymer with any functional groups compatible both with the functionalization step and with the fluorescent labeling.

The second strategy is based on the pre-branching functionalization (Scheme 1, bottom part). In this case, the introduction of the anchoring group for the fluorescent labeling into the CD-monomer occurs before the branching of the modified CD. This strategy offers advantages in terms of versatility since a large variety of procedures for the modification of the CD monomers are well described in the literature as well as the methods for characterization [18]. The introduction of the anchoring group onto the monomer can be the only modification on the CD scaffold or “preliminary” modification of the scaffold can occur in order to vary the properties of the monomer, such as solubility and complexing ability.

In terms of characterization of the intermediates, the first strategy results in a more complicated derivative since the modification processes are barely specific for the CD ring but involve the bridges and side chains derived from the branching agent having hydroxy moieties as well. With the second strategy the labeling groups are located only on the CDs.

### First strategy

The tagging of the epichlorohydrin branched β-CD polymer with the fluorescent moieties by preserving the neutral charge of the unlabeled CD units is based on the following three consecutive steps:

1. Introduction of the azido groups into the β-CD polymer.
2. Reduction of the azido groups to amino moieties.
3. Fluorescent labeling of the β-CD polymer based on the reaction between the amino moieties and the isothiocyanate form of the dye (rhodamine or fluorescein).

**Scheme 2:**
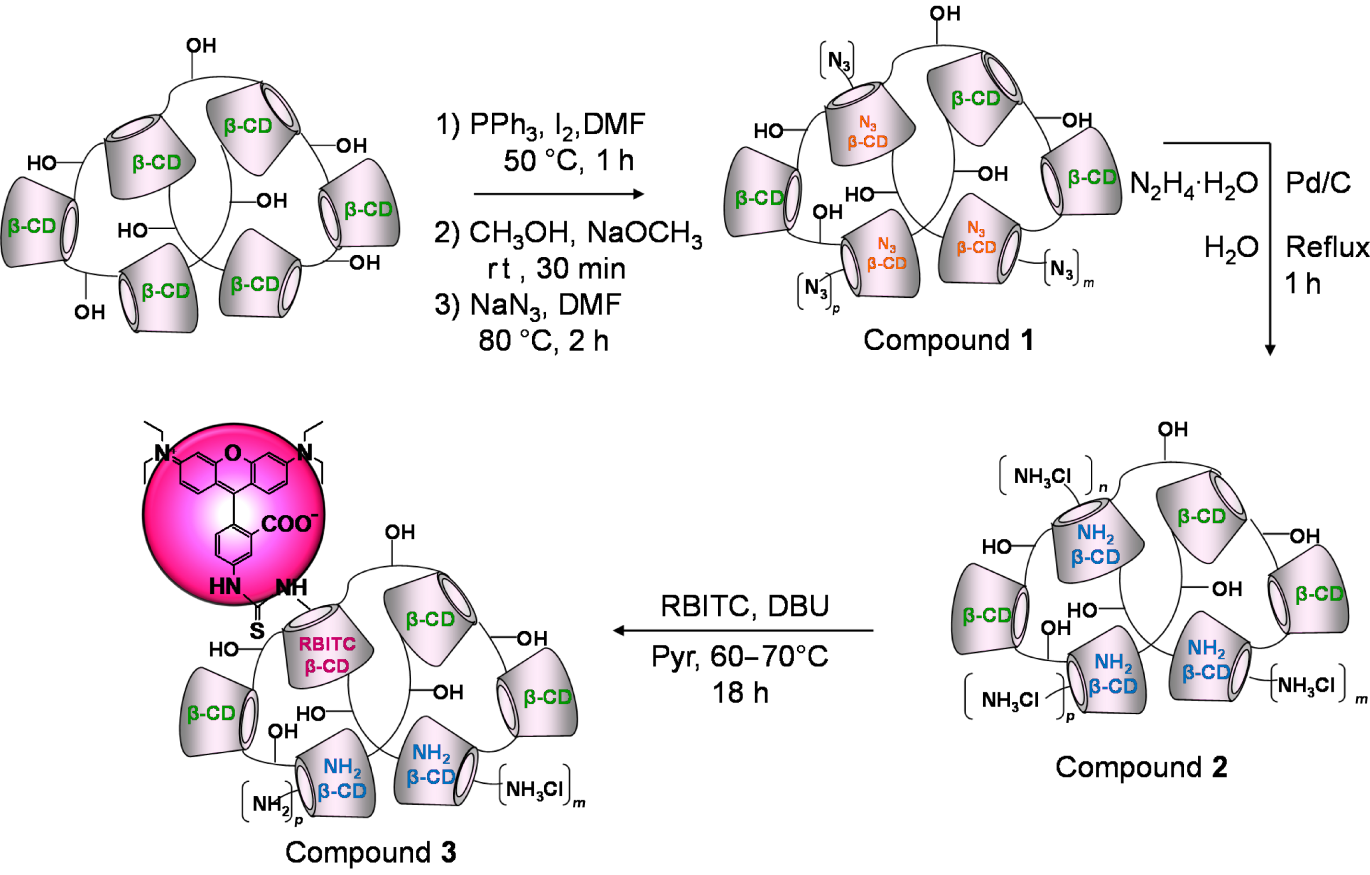
Synthetic strategy for the rhodaminylation of β-CD polymer.

As an example of the first strategy we report the fluorescent labeling of β-CD polymer with rhodamine isothiocyanate (Scheme 2).

#### 1) Introduction of the azido groups

**Figure 1:**
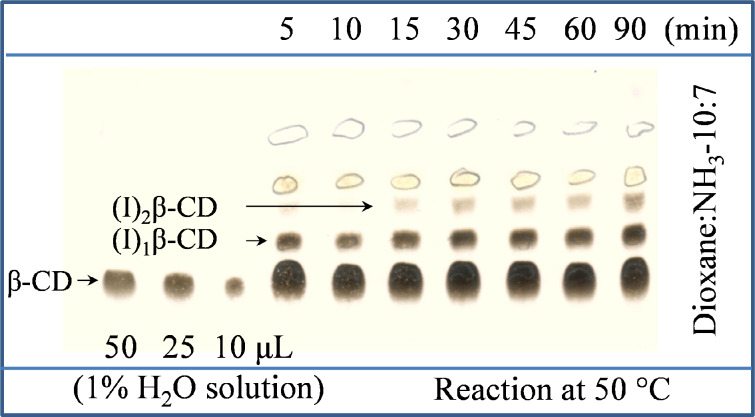
TLC study of β-CD iodination showing the proceeding of 6-monoiodination with increasing reaction time.

The introduction of the azido groups into the β-CD-polymer has been performed by adapting the sequence iodination → azidation [19] to the specific structure. The target was to introduce one azido moiety per CD ring in 0.5–2% of the CD population. The optimization of the three-step reaction conditions (Scheme 2) was performed on the corresponding monomer since the process can be easily followed by thin layer chromatography (TLC) in the case of the monomer while the polymer consisting of a mixture of components with wide range of molecular weight (*M*_w_) cannot be easily studied by TLC. According to our experience epichlorohydrin branched CD-polymers with relatively high *M*_w_ interact strongly with the stationary phase (normal silica, reverse-phase silica gel, alumina and so on) thus making it difficult to find a suitable eluent for removing the material from the baseline of the TLC plate. For the same reasons the work-up and the purification steps had to be carefully planned without relying on chromatographic techniques. Taken into consideration that the azidation step is an exhaustive process (if NaN_3_ is used in excess), the real challenge was to control the degree of the iodination. In the case of the β-CD the 60 min reaction was considered to be optimal for the 6-monosubstitution (Figure 1) and the identical conditions were used for the modification of the polymer.

**Figure 2:**
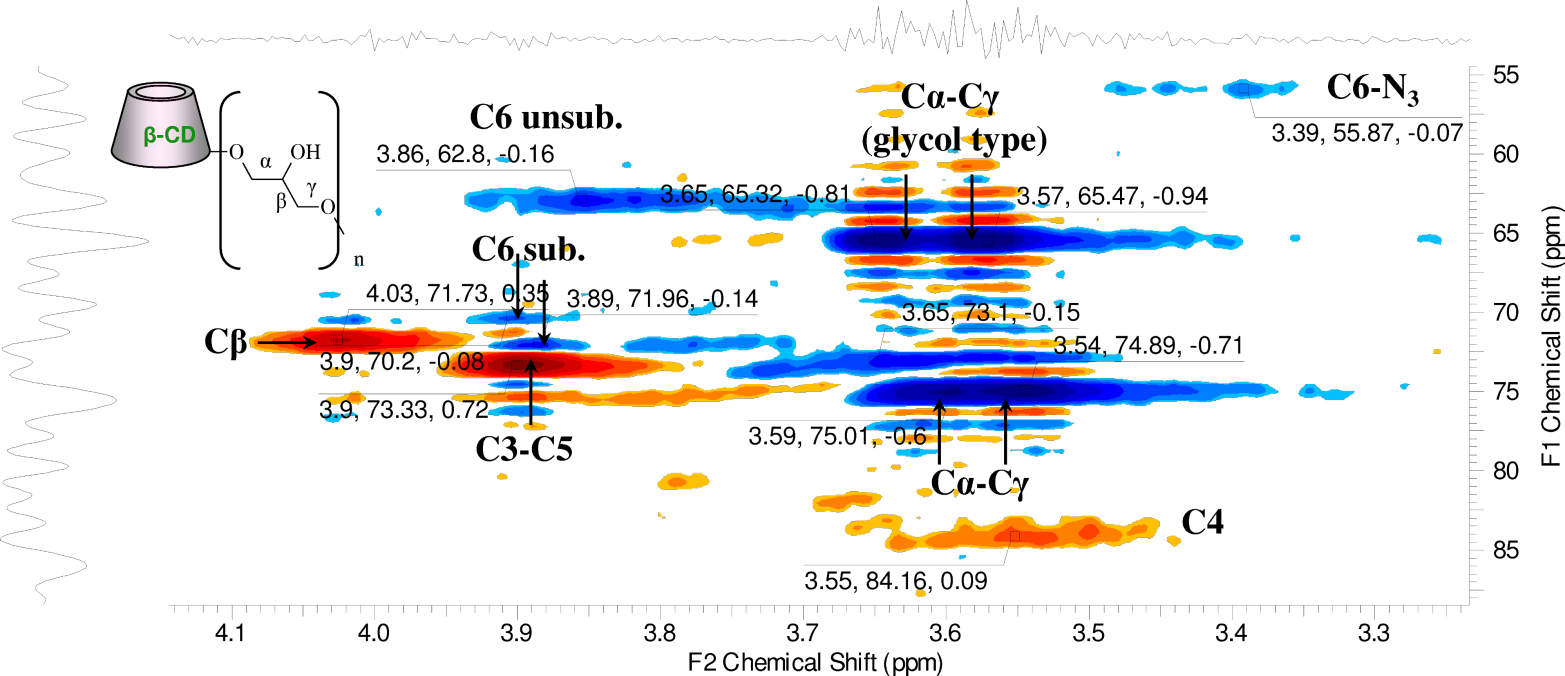
HSQC-DEPT spectrum of compound **1** with partial assignment.

In order to set the correct molar ratio between the reagents it is necessary (but not sufficient) to know the *M*_w_ of the polymer and its CD content. In spite of these parameters were clearly determined in the specific case (the *M*_w_ of the polymer was evaluated around 120 kDa and the CD content around 70% w/w) the introduction of one single azido unit on the primary side on 0.5–2% of the CD rings of the polymer was complicated by the impossibility to follow the progress of the reaction by TLC. The optimized reaction conditions for the azidation of the β-CD-polymer are shown in Scheme 2 while the work-up procedure is reported in detail in the experimental part (see Supporting Information File 1). Concerning the characterization of compound **1** (Scheme 2), a series of challenges related to the quantification of the introduced azido groups, has to be taken into account. The qualitative overall results of the reaction can be evaluated by IR owing to the detection of the azido peak (around 2103 cm^−1^, Supporting Information File 1, Figure S1), but the quantification of the azido groups in the polymer, the evaluation of the CD substituted/CD unsubstituted ratio and the determination of the azido groups per CD ring cannot be easily accomplished with standard methods. In theory, for the quantification of the total N_3_ groups in the polymer we could rely on the integration of ^13^C spectra. By dividing the integral of the C6–N_3_ substituted signals with that one of the anomeric carbon an estimation of total azido content in the polymer could be obtained. Indeed, from the HSQC-DEPT spectra of compound **1** (Figure 2), the C6–N_3_ is in a characteristic region of the spectrum (around 56 ppm) and it does not overlap with other signals.

The assignment of the C6–N_3_ signal can be done by cross-linking the HSQC-DEPT spectra of compound **1** and 6-monoazido-6-monodeoxy-β-CD (Supporting Information File 1, Figure S5); the assigments are consistent with the literature [20–21].

In conclusion, quantitative ^13^C spectra might be obtained by inverse gated decoupling and long delays in the region of 10 minutes between pulses. However, this expensive and time consuming method is very insensitive for ^13^C and is rarely a realistic option. A more efficient way to quantify the global amount of N_3_ groups in the polymer is based on the IR determination of the azido content as % w/w. The method relies on the possibility to produce a calibration curve that relates the amount of N_3_ (mg) to the absorbance of a specific region of the IR spectra (2350–1850 cm^−1^) (Supporting Information File 1, Figure S2). After calculation of the area of the band between 2350–1850 cm^−1^ for compound **1**, the amount of N_3_ was estimated to be around 0.6% w/w and the percentage of azidated-CDs in the polymer was calculated around 0.4% w/w.

Although it is possible to quantify the total amount of N_3_ in the polymer, the development of the method for the fine quantification of the azido groups per CD ring (since some CD can bear more than one N_3_ group) is still under investigation. The quantification of the azido groups per CD ring in a “randomly” azidated epichlorohydrin branched β-CD-polymer is a difficult task.

#### 2) Reduction of the azido groups to amino moieties

**Figure 3:**
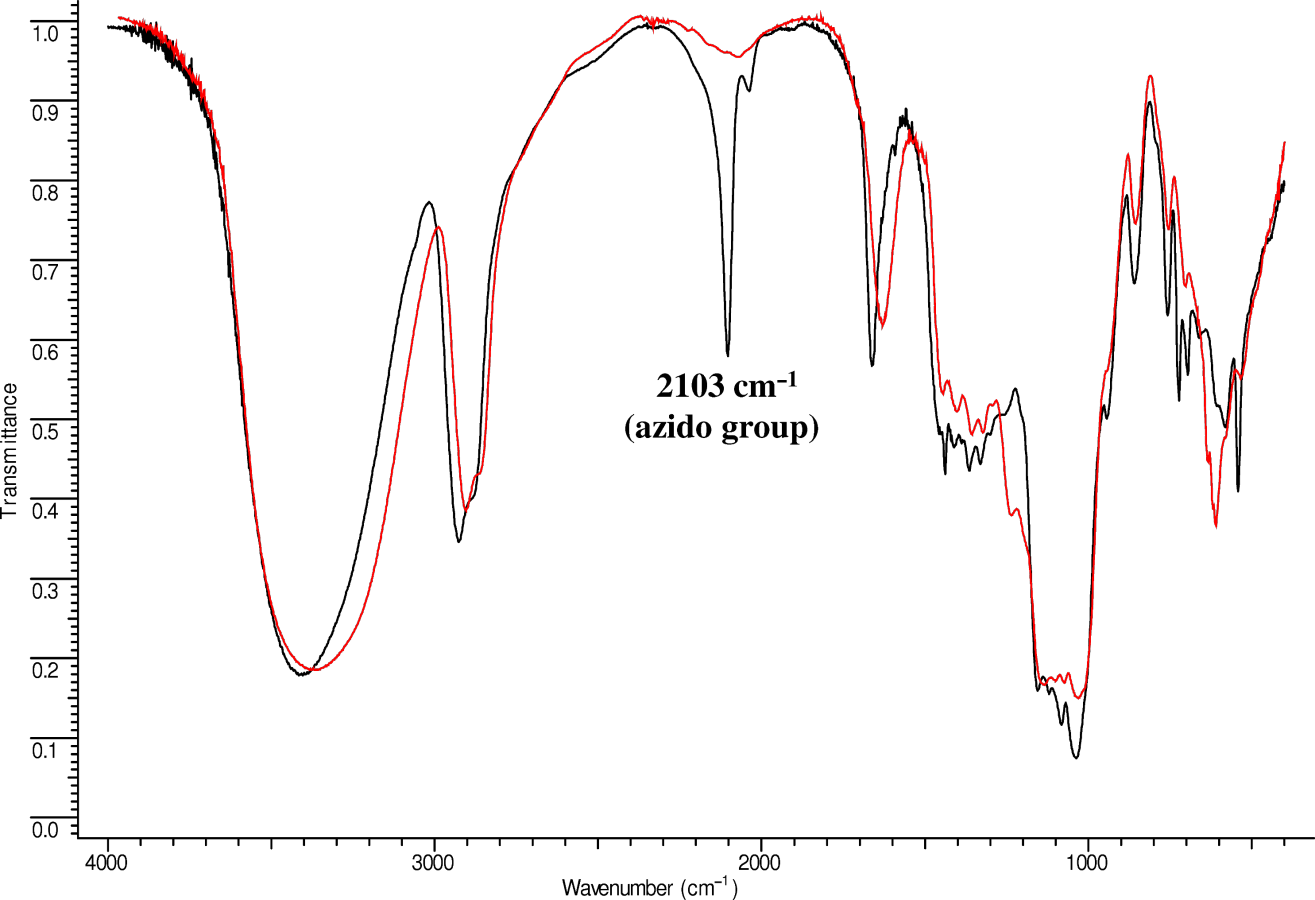
IR spectra of compound **1** (black line) and compound **2** (red line) showing the disappearance of the azido peak after reduction.

There are several and selective ways to perform the azido→amino conversion [22] and for compound **1** the hydrogenolytic method [23] based on hydrazine, catalyzed by Pd/C (Scheme 2) was selected. The reaction using hydrazine-Pd/C is fast and exhaustive (based on the evaluation of the IR spectra, Figure 3), but because of the laborious work-up (filtration, centrifugation, membrane filtration) and the partial inefficacy of the dialysis purification, this method needed further development.

The overall progress of the reaction can be estimated by IR since the intense azido peak completely disappears during the reduction: as an example the spectra for compound **1** before and after the reduction is shown in Figure 3.

The analysis of the HSQC-DEPT spectra of compound **2** (Supporting Information File 1, Figure S8) shows the disappearance of the C6–N_3_ signal around 56 ppm, but the identification of the expected new signals for the C6–NH_2_ (around 40 ppm) is rather troublesome and can be barely detected because of the unfavorable signal-to-noise ratio.

As a proof of the assignment of the C6–NH_2_ in the HSQC-DEPT spectrum of compound **2** (Supporting Information File 1, Figure S8), the HSQC-DEPT spectrum of per-6-amino-β-CD is reported for comparison in Supporting Information File 1, Figure S9. It is worth to emphasize that in this last spectrum the signal of the C6/unsubstituted is not present since all the primary methylene units are NH_2_-substituted, confirming in this way the structure of the compound. All the assignments are consistent with the literature [24]. The change in the molecular weight for compound **1** after the reduction is small, a complete reduction of the azido groups would decrease the average molecular weight only of around 2 kDa (1.4% of the starting weight).

#### 3) Fluorescent labeling

The coupling between rhodamine isothiocyanate (RBITC) and compound **2** was performed in pyridine and the addition of an extra base (DBU) contributed to the progression of the reaction (Scheme 2). As in the case of the fluorescent labeling of simple amino-β-CD monomers [25], the choice of the solvent and of the base can be fundamental for achieving the goal. The removal of the undesired fluorescent byproducts is a troublesome task since a selective precipitation cannot be accomplished and chromatography is not an effective purification technique due to the strong interaction between the stationary phase and the polymer. In this case liquid/liquid extraction is necessary to remove the bulk of the dye-related byproducts, the hot charcoal treatment can decrease their amounts and the final dialysis step (or ultrafiltration) can further reduce the impurities level.

The strategy that was built-up for achieving the labeling can introduce some undesired alteration into the polymeric structure. If the target is a strictly homogeneously fluorescent-labeled β-CD polymer, then the presence of unreacted amino groups, owing to an incomplete fluorescent labeling, could be an undesired modification of the starting material that can affect some properties such as the water solubility and/or ionizability. Furthermore, the quantification of these (eventual) residual amino groups would be troublesome.

**Scheme 3:**
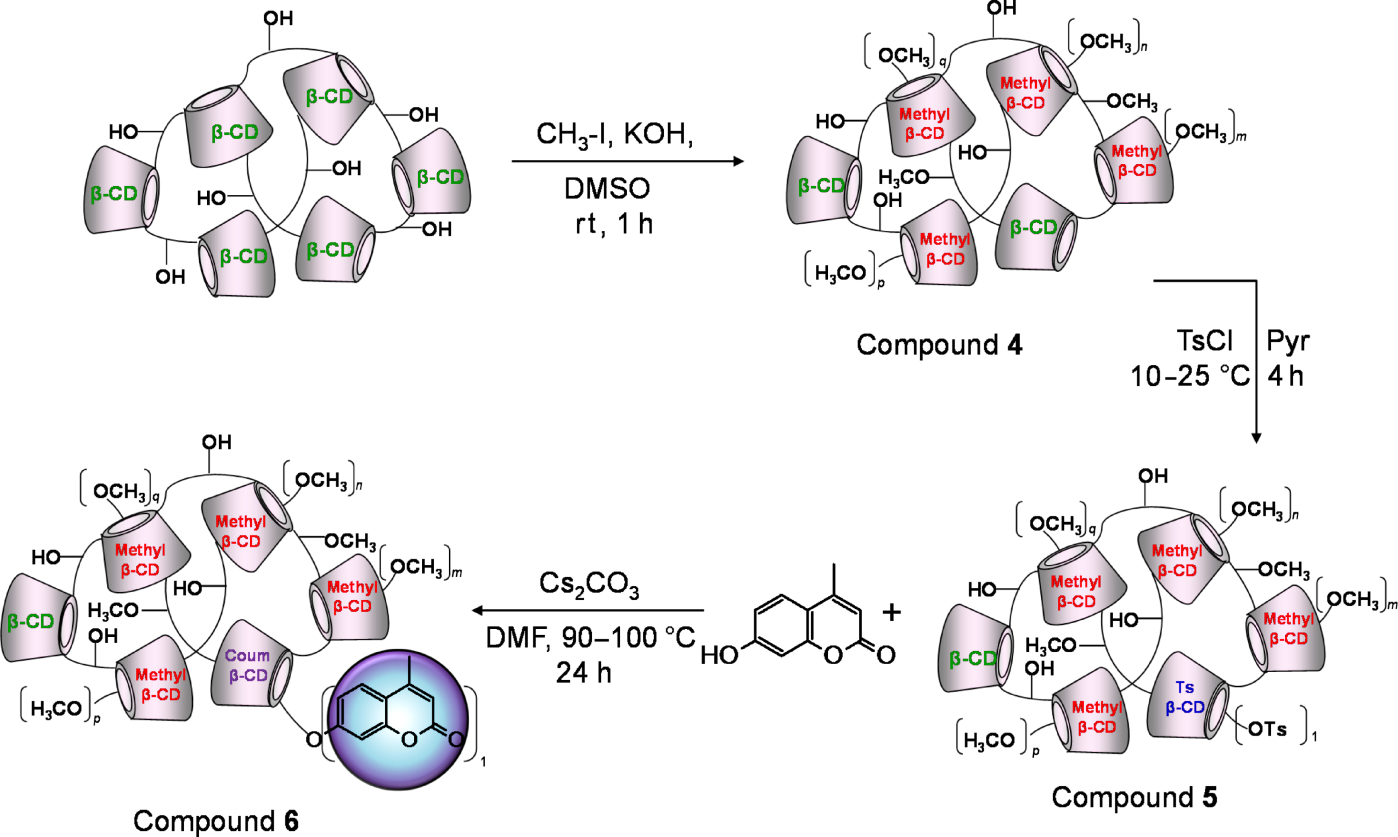
Schematic representation for the coumarinylation of methylated β-CD-polymer, *n*, *m*, *p* and *q* mean the number of methoxy groups on the individual CD units.

The strategy that was applied to overcome the problem of residual unreacted amino groups in the polymer was the use of an excess of the fluorescent dye (at least 2 fold the molar amount of the approximated amino groups), diluted solution for improving the solubility of the dye and long reaction time. The optimization of these reaction conditions can reasonably lead to a homogeneously fluorescent labeled polymer. The quantification of the (eventual) unreacted amino groups can be achieved by determining (by UV–vis and CE measurement) the amount of dye covalently bound to a weighed amount of fluorescent labeled polymer and by subtracting this value from the amount of amino groups in the starting compound **2**. The amount of initial NH_2_ groups in compound **2** can be derived from the azido content calculated by IR titration for compound **1**. In more detail, the initial NH_2_ content for compound **2** corresponds to 0.15% w/w, by considering a 100% conversion of the azido groups that is 1.5 mg (0.094 mmol) of NH_2_ group in 1 g of compound **2**. From the UV–vis measurement of compound **3**, the total amount of rhodamine was calculated to be around 0.5% w/w, that means 96 ± 4% of the amino groups reacted. By considering that the free dye content was determined to be around 0.01% w/w by capillary electrophoresis (CE), the amount of dye covalently attached to the polymer is between 0.45–0.5%. As previously mentioned, the strategy based on the post-branching modification (Scheme 1, upper part) can include a supplementary step for the modulation of the properties of the polymeric network before the introduction of the anchoring group for the fluorescent labeling (Scheme 3).

Methylated cyclodextrins have peculiar properties concerning both solubility and complex formation thus targeting a water-soluble methylated β-CD polymer with a low degree of substitution can be particularly important. As in the case of compound **1**, the setting and the optimization of the reaction conditions were performed for the corresponding monomer in order to achieve a methylated CD with an average degree of substitution around 4 (Supporting Information File 1, Figure S15). The optimized conditions were then used for the methylation of the polymer. The methylation in DMSO and KOH is effective at very mild conditions.

**Figure 4:**
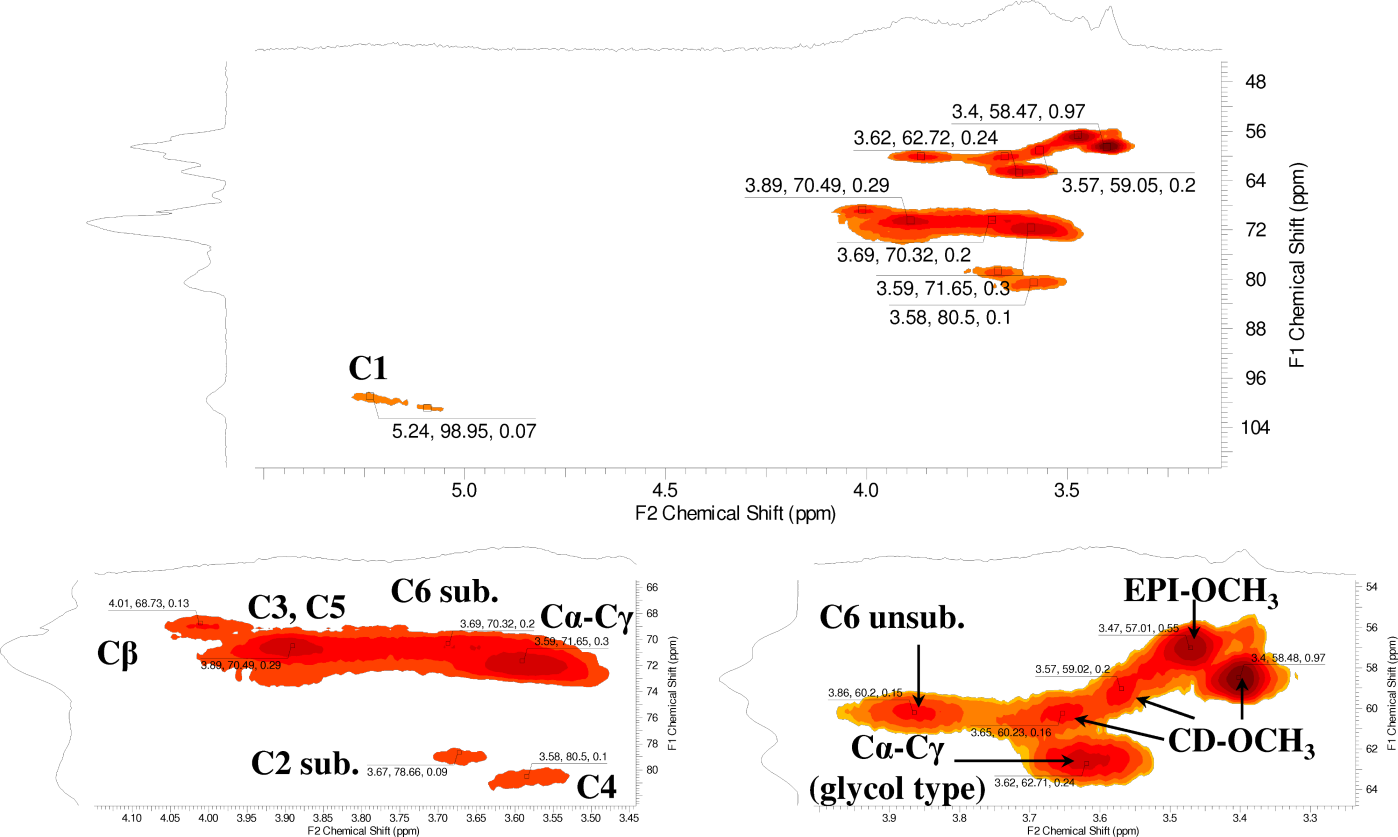
HSQC-DEPT spectra of compound **4** with partial assignment; in the upper part the full spectrum is shown and on the bottom some regions are zoomed.

As the process is a random reaction, the branching agent is methylated as well. By comparing the HSQC-DEPT spectrum of the methylated β-CD (Supporting Information File 1, Figure S14) with that one of the methylated β-CD polymer (Figure 4), the assignments of the signals for methyl groups can be accomplished. The signals that correspond to the methylated groups directly attached to the CD moieties resonate around 58–60 ppm and span from 3.35 to 3.65 ppm (Supporting Information File 1, Figure S14). The signal at (3.47, 57.01) ppm (Figure 4, EPI-OCH_3_) which is only present in the methylated polymer belongs to the methyl group attached to the branching agent.

The methylation of the branching agent affects the determination of the total amount of methyl groups calculated by ^1^H NMR since all the signals overlap in the region between 3.0–4.5 ppm (Supporting Information File 1, Figure S12). For the evaluation of the amount of methyl groups in compound **4** the CD content of the starting β-CD polymer (CD content ca. 70%) must be taken into consideration. The methylation process is assumed not to influence the molar ratio of the branching agent and the CD units and the total amount of methyl groups can be effectively determined by ^1^H NMR (Supporting Information File 1, Figure S12). According to this approach the number of methyl groups was calculated as about 5.6 per CD unit. The determination of the molecular weight is in progress, we have to find the proper solvent as in water unreliable values were obtained.

The following modification of the methylated polymer is the introduction of the dedicated anchoring group for the fluorophore, which is in the specific case a tosyl moiety (Scheme 3). Because both the methylation and the branching affects almost exclusively the secondary hydroxy groups, free primary hydroxy groups are accessible for tosylation. The tosylation of CD derivatives as well as the work-up of the obtained crude compounds are well described in the literature [26]. The selected procedure for the tosylation of the methylated polymer is an adaptation of the classical procedure to the specific structure. Concerning the characterization of compound **5**, the methyl and tosyl content can be determined by comparing ^1^H NMR spectra of the starting material with that one of the product as shown in Supporting Information File 1, Figure S16.

The tosyl-related impurities (tosic acid/tosylate) were determined by CE and the level of the impurities resulted below 0.02% w/w. The introduction of tosyl groups into the methylated polymeric network only slightly modifies the molecular weight of the polymer compared to the starting methylated polymer (see experimental part in Supporting Information File 1). Although the reaction is performed in diluted pyridine and the TsCl is in large defect, the substitution should mainly occur on the primary side of the CD; unfortunately, this assumption could not be verified by the analysis of the HSQC-DEPT spectrum (Supporting Information File 1, Figure S17). The signal of the C6-tosyl substitution could not be detected due to the low amount of tosyl introduced into the polymer and to the lower sensitivity of the HSQC-DEPT experiment compared to the simple monodimensional ^1^H NMR. The C6-tosyl substituted moiety should resonate around (4.2, 70.2) ppm as shown in the HSQC-DEPT spectrum of 6-monotosyl-β-CD (Supporting Information File 1, Figure S18).

The introduction of the 4-methyl-7-hydroxy-coumarinyl moiety into the polymer was achieved by adapting a previously described synthetic strategy [27] to the macromolecular environment. The work-up and the purification processes are laborious, the removal of the undissolved Cs_2_CO_3_ requires repeated centrifugation cycles and for the complete removal of the untagged 4-methyl-7-hydroxy-coumarin a series of liquid–liquid extraction steps together with a dialysis cycle must be applied. The CD content of compound **6** determined by ^1^H NMR is around 70% w/w and the molar ratio between the methyl groups and the CD units is 4.8 (Supporting Information File 1,Figure S19). The substitution of tosyl groups with the 4-methyl-7-hydroxycoumarinyl moieties only slightly modifies the molecular weight of the polymer compared to the starting material according to the SLS measurements (see experimental part in Supporting Information File 1,). The molar ratio between the 4-methyl-7-hydroxycoumarinyl groups and the CD units was determined by ^1^H NMR with the usual integration method and it was estimated as 0.12 (Supporting Information File 1, Figure S19). The amount of untagged coumarin was determined by CE and was evaluated less than 0.01% w/w. The (eventual) unsubstituted tosyl groups were quantified by ^1^H NMR since the signals of the aromatic protons of the tosyl resonate in a separate region of the spectrum (between 7.0–8.0 ppm) (Supporting Information File 1, Figure S16). For compound **6** no residual tosyl groups were detected by ^1^H NMR.

### Second strategy

**Scheme 4:**
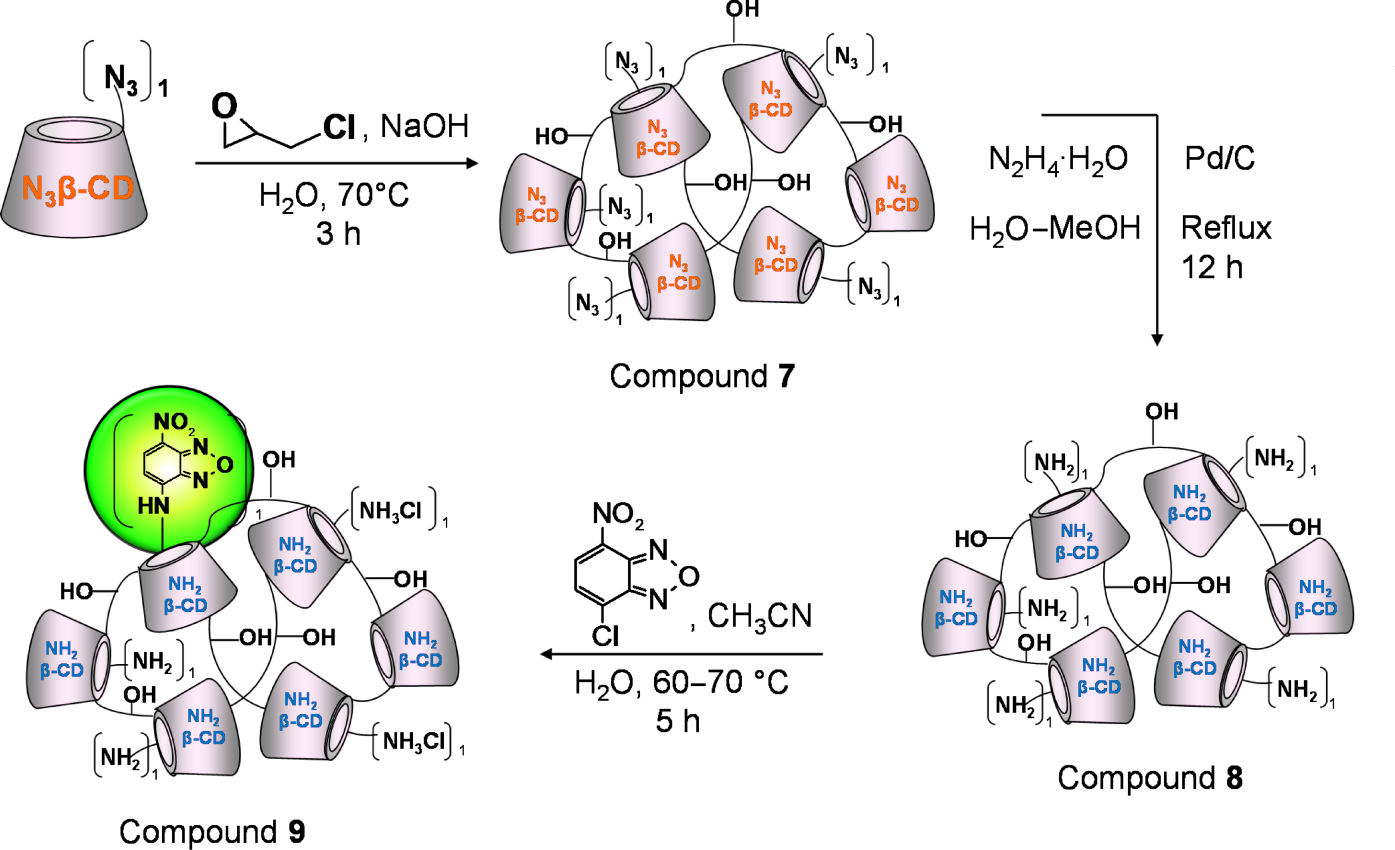
Schematic representation for the introduction of NBF in a cationic β-CD-polymer.

The nitrobenzofurazanyl (NBF) tagging of the epichlorohydrin branched cationic β-CD polymer reported in Scheme 4 is an example of pre-branching modification.

According to this strategy the anchoring group for the fluorescent tagging is introduced into the CD monomer before the branching in the form of azido group as precursor for the amino moiety. The eventual modification of the 6-monoazido-6-monodeoxy-β-CD monomer with pendant group that do not interfere with any of the steps for the fluorescent tagging can add interesting properties to the polymeric network.

The branching of 6-monoazido-6-monodeoxy-β-CD (Scheme 4) with epichlorohydrin leads to an azidated polymer that does not present the characterization problems described for compound **1**. Indeed the quantification of the azido groups per CD ring does not make difficulties since each CD (azido-substituted) bears only one azido moiety at the primary side. The CD content of the azidated polymer was measured by ^1^H NMR (Supporting Information File 1, Figure S22) and the N_3_ content was calculated to be around 2.4% w/w. The molecular weight of this polymer as determined by SLS is a lower value compared to that one obtained for compound **1**. Since the branching conditions, the work-up procedure and the purification process are similar as in the case of a simple β-CD polymer it can be argued that the lower molecular weight is the result of the mono-modification on the 6-position of the CD monomer as this is the main target also for the reaction with epichlorohydrin. At this regard two parameters can be taken into considerations: the lower number of hydroxy groups per CD ring available for the branching and the reduced solubility of the 6-monoazido-6-monodeoxy-β-CD in alkaline conditions compared to the β-CD, both influencing negatively the degree of branching.

The reduction of compound **7** resulting in compound **8** proceeds smoothly as in the previous case (compound **2**). The work-up, the purification and the considerations about the characterization are similar to those as for compound **2**.

The labeling of the cationic polymer with NBF is based on the nucleophilic substitution between the amino groups and NBF-Cl. Nitrobenzofurazan is a green dye that is well-known in the field of peptide chemistry since it was first applied as a fluorogenic reagent for amino acids [28]. The high reactivity of the 4-chloro-7-nitrobenzofurazan towards amines and its peculiar spectroscopic properties make this molecule a convenient reagent for selected experiments. This fluorophore reacts promptly with free amino groups and shows fluorescence only when substituted with amino moiety. Taking in consideration that finding an appropiate eluent to make a CD-based polymer run on a TLC plate is a challenging task, having a reagent that shows fluorescence only if the reaction is successful is an undeniable advantage. In fact, this reaction, although the starting material and the product stuck on the baseline of the plate, can be followed by TLC due to the easy detectability of the amino-substituted fluorophore. As can be seen in the TLC in Supporting Information File 1, Figure S30, the spot on the baseline is fluorescent under UV light at 366 nm and simultaneously charrable thus indicating the presence of CD units.

The purification of compound **9** can be accomplished by taking advantage of the high solubility of the unreacted NBF-Cl in DCM (one cycle DCM/water extraction is sufficient to remove all the unreacted dye) and by performing a dialysis step to remove the traces of the organic solvent.

This fluorescent cationic polymer was characterized by spectroscopic and chromatographic techniques. From the ^1^H NMR spectrum (Supporting Information File 1, Figure S28) the molar ratio between the dye and the CD units and the CD content of the polymer were determined as 0.12 and 68% w/w respectively. The NBF moiety presents 2 broad singlets in 2 different regions of the spectrum: one between 6 and 7 ppm that can be assigned to the proton in ortho position with respect to the amino group and the second one more downfield between 8 and 9, which is assigned to the proton in ortho to the nitro group. By setting to 7 the integral of the anomeric region, the molar ratio between the CD and NBF was calculated (DS ca. 0.12 in Figure S28, Supporting Information File 1).

**Scheme 5:**
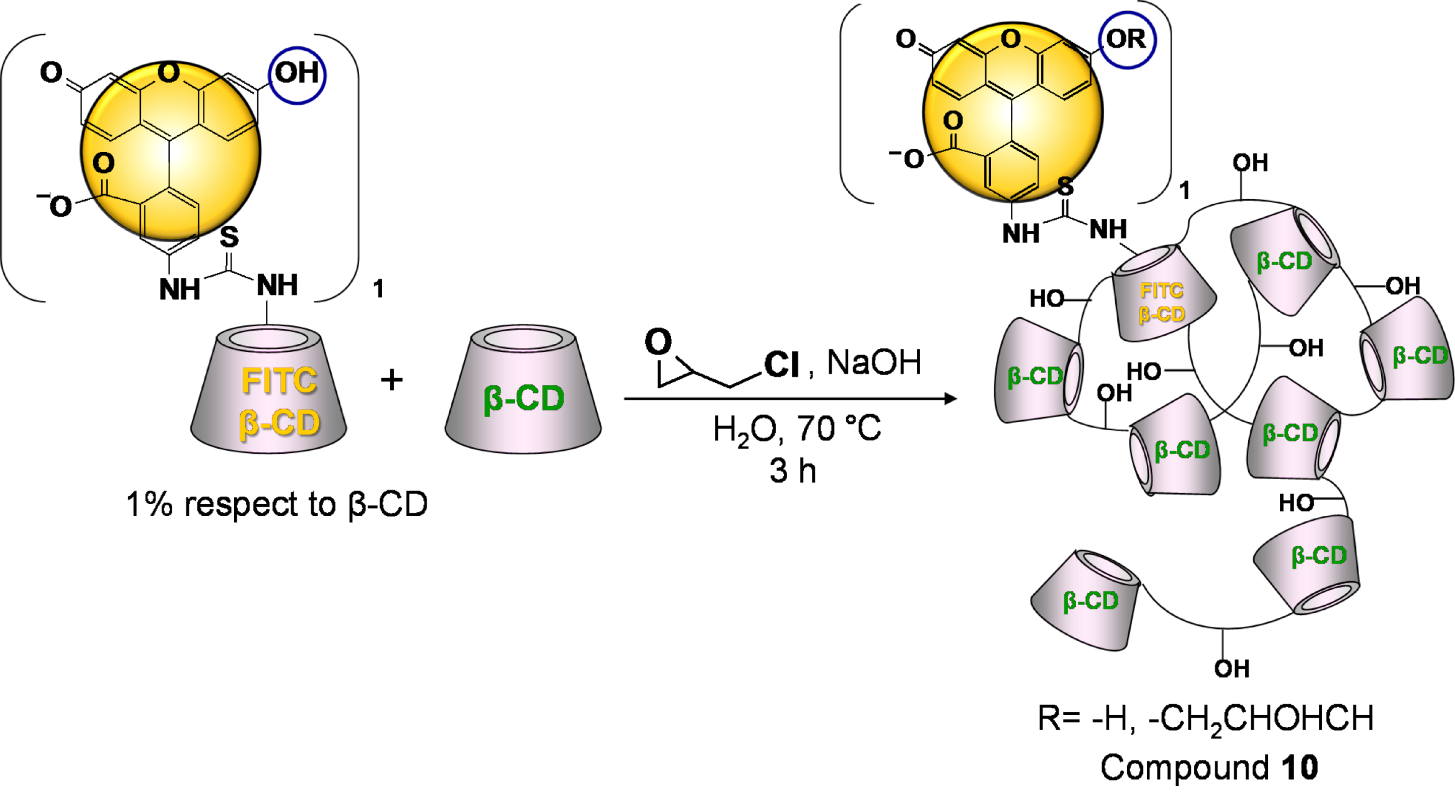
Schematic representation for the introduction of fluorescein into a β-CD-polymer.

From the ^13^C spectrum of compound **9** (Supporting Information File 1, Figure S29) the signal of the C6-NH_2_ substituted (around 40 ppm) was detected confirming that free amino groups remain in the polymer after the labeling. The content of the untagged NBF was evaluated by TLC and the amount is less than 0.1% w/w (Supporting Information File 1, Figure S30). A slight deviation from the strategy based on pre-branching modification is represented in Scheme 5.

According to this approach the fluorescent dye is introduced into the β-CD monomer [29] and the resulting fluoresceinyl β-CD (FITC-β-CD) is mixed in the desired ratio with native β-CD before the branching. During the branching process, part of the FITC-β-CD is incorporated into the polymeric network and after neutralization of the crude and dialysis a fluorescent β-CD polymer is obtained (compound **10**).

This approach has some advantages compared to the procedure described in Scheme 2 (post-branching modification) since the purification, the characterization and the assessment of the purity for the FITC-β-CD are well established procedures compared to the corresponding polymer. Furthermore, as the thioureido linkage is stable under the branching conditions, no free dye is generated during the reaction thus simplifying remarkably the work-up. It is worth to remind that the removal of the excess of the dye from the crude of the reaction is always a primary and challenging task.

The strong alkaline conditions set for the branching can lead to a phenolate moiety able to react with the branching agent thus generating a (2-hydroxy)propyl-substituted aromatic ether (Scheme 5, blue circles). The (eventual) modification of the fluorescein at the phenolic hydroxy group during the branching step preserves the fluorescence of the molecule as previously described by Ueno [30]. Concerning the characterization of the compound, the CD content and the molar ratio of the fluorescein with the CD units were determined by ^1^H NMR analysis (Supporting Information File 1, Figure S32) (69% w/w, 0.02 respectively). Qualitative information about the pattern of substitution was gained by interpretation of the HSQC-DEPT spectrum of the macromolecule as can be seen by the partial assignment in Figure S33. The molecular weight was estimated by SLS around 40 kDa and the free fluorescein content was determined by CE below 0.01% w/w. All the characteristics of compound **10** are reported in the experimental part in Supporting Information File 1.

## Conclusion

Two versatile synthetic strategies for obtaining the fluorescent labeled epichlorohydrin branched β-CD polymer were built-up: i) modifying the epichlorohydrin branched polymer or ii) modifying the monomer prior to branching. The strategies were demonstrated on several examples using various fluorescent moieties for labeling.

For the first strategy, the fluorophore is attached on the primary side of the CD scaffold, if the sequence iodination→azidation is used. If the intermediate product is a tosylated polymer, the amount and the distribution of fluorophore depend strictly from the tosylation step. If tosyl chloride is used in defect in respect to the amount of CD units, the substitution occurs preferentially on the primary side, if used in larger amounts (i.e., 2–3 mol per CD ring) the substitution can involve the secondary side of CD as well.

In the secomd strategy, the fluorescent labeled β-CD monomer is polymerized together with native β-CD. The fluorophore is always (and only) on the primary side, since the fluorescent labeling for the monomer is performed through 6-monoamino-6-deoxy-β-CD.

The structures of the polymers were characterized by NMR and IR spectroscopies, their molecular weight was determined by SLS and the purity was checked by CE. The syntheses of amino- and tosyl-modified β-CD polymers as intermediates allow the introduction of various electrophilic (i.e., RBIT, NBF-Cl, FITC) or nucleophilic fluorescent dye (i.e., coumarin) into the polymers, respectively. The key intermediate azido-modified polymers have been used as precursor of the amino-modified polymers, but they can be considered as versatile substrates for cycloaddition reactions as well. Both synthetic strategies allow the introduction of additional pendant groups into the polymeric structures thus enriching the properties of the targeted compound, if needed; this process is demonstrated by the methylation of the epichlorohydrin branched β-CD polymer.

The feasibility of the strategies was demonstrated for β-CD, but they can be easily adapted for the production of α- and γ-CD polymers as well. The applications of fluorescent labeled epichlorohydrin branched β-CD polymers span from the biological field and material science to the pure physicochemical investigations.

## Supporting Information

File 1 Experimental part, IR, ^1^H, ^13^C or HSQC-DEPT spectra of the synthesized compounds.
