# Supplementary material for: Synthetic strategies for the fluorescent labeling of epichlorohydrin-branched cyclodextrin polymers
Source: Beilstein J Org Chem. 2014 Dec 16;10:3007–18. doi: 10.3762/bjoc.10.319 (PMC4311710; doi:10.3762/bjoc.10.319)
Supplement: File 1 — Experimental part, IR, 1H, 13C or HSQC-DEPT spectra of the synthesized compounds. [file Beilstein_J_Org_Chem-10-3007-s001.pdf]

Supporing Information

for

Synthetic strategies for the fluorescent labeling of epichlorohydrin-branched cyclodextrin polymers

Milo Malanga[*], Mihály Bálint, István Puskás, Kata Tuza, Tamás Sohajda, László Jicsinszky, Lajos Szente, Éva Fenyvesi

Address: CycloLab Cyclodextrin Research and Development Laboratory Ltd, Illatos 7, Budapest, H-1097 Hungary.

Email: Milo Malanga - malanga@cyclolab.hu

*Corresponding author

Experimental part, IR, ^{1}H, ^{13}C or HSQC-DEPT spectra of the synthesized compounds

Experimental

Material and Methods

Epichlorohydrin branched β-CD water soluble polymer (poly-β-CD, molecular weight 120 ± 5 kDa), 6-monoazido-6-monodeoxy-β-CD and 6-deoxy-6-[(5/6)-fluoresceinylthioureido]-β-CD (FITC-β-CD) are fine chemical products of CycloLab. Rhodamine B isothiocyanate mixed isomers (RBITC), hydrazine monohydrate (98%), triphenylphosphine (99%), iodine (99.8%), cesium carbonate (99%), tosyl chloride (99%), iodomethane (99.5%), 7-hydroxy-4-methyl-coumarin (98%, CMR), (±)-epichlorohydrin (99%), 4-chloro-7-nitrobenzofurazan (98%, NBF-Cl), potassium hydroxide (85%), sodium methoxide (97%), sodium azide (99.5%), dichloromethane (DCM), dimethylformamide (DMF), pyridine (Pyr), dimethylsulfoxide (DMSO) and acetonitrile were purchased from Sigma-Aldrich; 1,8-diazabicyclo[5.4.0]undec-7-ene (99%, DBU) was purchased from Fluka; palladium/charcoal activated (10% Pd) was purchased from Merck.

Charcoal activated (puriss.) was a product of Molar.

Membrane filters (0.45 μm, 47 mm diameter, cellulose acetate) were from Sartorius Stedim.

All the reagents were used without further purification. Solvents were dried by conventional methods and distilled immediately prior to use.

Dialysis tube cellulose membrane molecular weight cut-off= 14.000 was from Sigma.

Silica gel coated aluminum sheets were from Merck (Art. No.: 1.05554). Plates were developed in a saturated chamber in a 1,4-dioxan:ammonium hydroxide (25%) = 10:7 (v/v) or abs. ethanol:water = 4:1solvent systems. Visualization was achieved under UV light at 254/366 nm by charring with a solution of EtOH (96%):H_2SO_4 (96%) = 9:1 charred by heating at 105-110 °C.

^{1}H-, ^{13}C- NMR spectra and HSQC-DEPT spectra were recorded on a Varian VXR-600 at 400 or 600 MHz. UV/Vis absorption spectra were recorded with a Hewlett Packard 8452A

spectrophotometer. IR spectra were recorded in KBr disk on a Nicolet 205 FTIR spectrometer. CE experiments were conducted on an Agilent 7100 Capillary Electrophoresis System equipped with Diode Array Detector (Waldbronn, Germany).

Molecular weight determination was performed using a Malvern Zetasizer Nano ZS instrument running a Static Light Scattering (SLS) method.

The viscosities of 1.00 w% solutions were determined by a Cannon-Fenske viscometer at 20.0 °C.

The total fluorescent dye content was evaluated by ^{1}H-NMR and/or by UV-Vis spectroscopy. The latter estimation was performed based on a calibration curve using as standard the starting fluorescent dye (RBITC, NBF-Cl or CMR) or the fluorescent-labeled β-CD monomer (FITC-β-CD) in the case of compound **10**.

The free dye content was measured by CE in 30 mM NaH_2PO_4 buffer of pH set to 6.1. The samples were run in uncoated fused silica capillaries of 25 cm effective length at 20 kV applied voltage and introduced hydrodynamically at 200 mbar·s. On each day, before a set of measurements the capillary was washed with water for one minute, followed by 1 M NaOH for ten minutes, 0.1 M NaOH for three minutes and again water for one minute. Between the runs, the capillary was flushed with 0.1 M NaOH-water-0.1 M NaOH for 1 minute each and with the operating buffer for 2.5 minutes. A series of calibration solutions corresponding to 5-0.2% of free dye in the product were applied for quantification.

The CD content for the polymers was determined by ^{1}H-NMR spectroscopy as reported in [1]. The azido content (w/w) for compound **1** was evaluated by IR. A calibration curve was built-up by relating increasing amount of NaN_3 weighed under inert atmosphere to the absorbance of the IR spectra between 2350-1850 cm^{-1}. By linear regression (R^2=0.990) it was possible to determine the amount of N_3 by knowing the area of the band in the specific region according to the following equation:

Area= 270.41 · N_3 amount (mg).

After calculation of the area of the band between 2350-1850 cm^{-1} for compound **1**, the amount of N_3 was estimated to be around 0.6 % (w/w).

Once determined the amount of N_3 in the polymer and the CD content (for compound **1** around 72% w/w), the percentage of azidated-CDs in the polymer can be calculated as follows:

Azidated CDs (%)= (72 · 0.6)/100=0.4 % (w/w).

The content of amino groups for compound **2** was calculated by assuming the exhaustive reduction of the azido groups of compound **1** as determined by the aforementioned IR method. The molecular weight for all the polymers was determined by SLS in water as previously reported in [2].

Synthesis of fluorescent epichlorohydrin branched CD polymers

(6-Azido-6-deoxy)$_n$-poly-β-CD **(1)**

Ph_3P (16 mg, 0.06 mmol) was dissolved in dry DMF (20 mL) and I_2 (16 mg, 0.06 mmol) was added by keeping the temperature of the reaction mixture below 40 °C. Dried poly-β-CD (2.1 g) was added to the dark brown solution, the temperature was increased to 50 °C and the mixture was stirred for 1 h. The solution was cooled down to 30 °C, CH_3OH (100 mL), $NaOCH_3$ (0,1 g, 1.85 mmol) were added and the mixture was stirred for 30 min. Methanol was removed from the suspension under reduced pressure (T=40 °C), DMF (~2 mL), NaN_3 (8 mg, 0.12 mmol) were added and the solution was heated at 80 °C for 2 h. Solvents were removed under reduced pressure (T=80 °C) and addition of water (20 mL) resulted in precipitation of a white solid. The solid was filtered off, the filtrate was extracted with dichloromethane (40 mL) and the aqueous phase was dialyzed over deionized water for 24 h. Freeze drying of the dialysate yielded compound **1** as white powder (1.9 g).

IR (KBr) ν/cm^{-1}: 3404 (O-H), 2927 (C-H), 2103 (azido), 1664, 1155, 1121, 1083, 1038, 859, 757, 723, 696, 542.

Assignments based on HSQC-DEPT (D_2O): δ= (proton, carbon) X, (3.39, 55.87) C6-N_3, (3.54, 74.89) Cα-Cγ, (3.55, 84.16) C4, (3.57, 65.47) Cα-Cγ (glycol type), (3.59, 75.01) Cα-Cγ, (3.65, 65.32) Cα-Cγ (glycol type), (3.86, 62.80) C6 unsub., (3.89, 71.96) C6 sub., (3.90, 70.20) C6 sub., (3.90, 73.33) C3-C5, (4.03, 71.73) Cβ, (5.04, 104.64) C1.

CD content based on ^{1}NMR: 72 % (w/w).

N_3 content based on IR calibration: 0.6 % (w/w).

Molecular weight based on SLS: 122 ± 3 kDa.

(6-Amino-6-deoxy)$_n$-poly-β-CD **(2)**

Compound **1** (1.9 g) was dissolved in H_2O (15 mL). First Pd/C suspension (0.18 g, 10% Pd content in 2 mL H_2O), then hydrazine hydrate (1 mL, 0.02 mol) was added and heated to reflux for 1 h. The reaction mixture was cooled to room temperature, the most of the charcoal was removed with a glass filter, the mother liquor was centrifuged, the supernate was membrane filtered and dialyzed over deionized water for 24 h. The pH of the solution was adjusted between 4 and 5 with HCl (1 N) and freeze-drying yielded compound **2** as a white powder (1.8 g).

IR (KBr) ν/cm^{-1}: disappearance of the azide band (2103), 3404 (O-H), 2927 (C-H), 1664, 1147, 1040, 861, 759, 620.

Assignments based on HSQC-DEPT (D_2O): δ= (proton, carbon) X, (3.32, 39.88) C6-NH_2, (3.51, 74.72) Cα-Cγ, (3.53, 84.10) C4, (3.56, 65.45) Cα-Cγ (glycol type), (3.59, 75.01) Cα-Cγ, (3.63, 64.32) Cα-Cγ (glycol type), (3.85, 62.80) C6 unsub., (3.87, 72.01) C6 sub., (3.92, 70.22) C6 sub., (3.89, 73.45) C3-C5, (4.03, 71.73) Cβ, (5.02, 103.53) C1.

CD content based on ^{1}NMR: 71 %(w/w).

NH_2 content (calculated) = 0.2 % (w/w).

Molecular weight based on SLS: 124 ± 8 kDa.

(6-Amino-6-deoxy)$_n$-6-[(5/6)-rhodaminylthioureido]$_m$- poly-β-CD **(3)**

Compound **2** (0.8 g) was dissolved in pyridine (10 mL). First DBU (10 mg, 10 μL, 0.05 mmol) then RBITC (6 mg, 0.01 mmol) were added and the solution was heated at 60-70 °C for 18 h. The solvent was evaporated under reduced pressure (T=60 °C), the crude material was dissolved in H_2O (50 ml) and extracted three times with DCM (100 mL). The aqueous phase was evaporated till dryness, the solid was dissolved in H_2O (50 mL) and extracted three times with ethyl acetate pre-saturated with water (100 mL). The aqueous phase underwent hot charcoal treatment, it was dialyzed for 24 h over deionized water three times and freeze drying yielded compound **3** as a violet powder (0.6 g).

IR (KBr) ν/cm^{-1}: 3410 (O-H), 2926 (C-H), 1652, 1593, 1155, 1082, 1041, 859, 757, 583.

In the ^{1}H-NMR spectrum (D_2O) the peaks of the methyl groups of rhodaminyl moiety appear at 1.25 ppm as a broad signal.

CD content based on ^{1}NMR: 69 % (w/w).

Total RBITC content based on ^{1}NMR: mol (RBITC) / mol (CD) = 0.12.

Total RBITC content based on UV-Vis calibration= 0.05 % (w/w).

Free rhodamine content based on CE measurement: < 0.01 % (w/w).

Molecular weight based on SLS: 123 kDa ± 12.

(Methyl)$_n$-poly-β-CD **(4)**

Dried poly-β-CD (10.26 g) was dissolved in DMSO (90 mL), KOH (13.5 g, 0.24 mol) was added and the solution was stirred at r.t. for 10 min. CH_3-I (2.8 mL, 45 mmol) was added and the solution was stirred at r.t. for 1 h. The crude was filtered, the mother liquor was

centrifuged and the supernate was decanted. Acetone (500 mL) was added to the supernate and a white soft material precipitated. The mixture was centrifuged and the supernate was removed by decantation. The solid was dissolved in water and dialyzed against deionized water for 48 h. Freeze drying yielded compound **4** as a white powder (6.17 g).

Assignments based on HSQC and DEPT spectra (D_2O): δ= (proton, carbon) X, (3.40, 58.48) CD-OCH_3, (3.47, 57.01) EPI-OCH_3, (3.57, 59.02) CD-OCH_3, (3.58, 80.50) C4, (3.59, 71.65) Cα-Cγ, (3.62, 62.71) Cα-Cγ (glycol type), (3.65, 60.23) CD-OCH_3, (3.67, 78.66) C2 sub., (3.69, 70.32) C6 sub., (3.86, 60.20) C6 unsub., (3.89, 70.49) (C3,C5), (4.01, 68.73) Cβ, (5.24, 98.95) C1.

The CD content was assumed as in the starting material β-CD polymer: 70 % (w/w) corresponds to the integral value of 77.89 protons between 3.0-4-5 ppm.

Total methyl content based on ^{1}NMR: mol (CH_3) / mol (CD) = 5.6

Viscosity of a 1% w solution: 1.125 cPoise.

*(Tosyl)$_m$-(methyl)$_n$-poly-β-CD)***(5)**

Dried compound **4** (1 g) was dissolved in pyridine (25 mL) and the solution was cooled down to 10 °C with water-ice bath. TsCl (0.3 g, 1.57 mmol) was added at once into the reaction mixture and the solution was allowed to warm-up to r.t. during 1 h. The solution was stirred for additional 3h. The crude was evaporated to dryness under reduced pressure (T=25 °C) till dryness, the gel-like residue was dissolved in H_2O (5 mL) and dialyzed against deionized water for 48 h. Freeze drying yielded compound **5** as a white powder (0.86 g).

In the ^{1}H-NMR spectrum (D_2O) the peaks of the aromatic proton of the tosyl groups appear between 7.5-8.0 ppm as two broad signals while the peak of the methyl group of the tosyl moiety appear around 2.5 ppm as a broad signal.

Assignments based on HSQC and DEPT spectra (D_2O): δ= (proton, carbon) X, (3.39, 58.52) CD-OCH_3, (3.46, 56.99) EPI-OCH_3, (3.51, 58.24) CD-OCH_3, (3.56, 58.97) CD-OCH_3, (3.55, 80.22) C4, (3.58, 72.19) Cα-Cγ, (3.61, 62.53) Cα-Cγ (glycol type), (3.66, 78.92) C2 sub., (3.67, 70.33) C6 sub., (3.86, 60.16) C6 unsub., (3.89, 70.38) (C3,C5), (4.00, 68.75) Cβ, (5.08, 100.06) C1.

The CD content was assumed as in β-CD polymer: 70 % (w/w) corresponds to the integral value of 77.89 protons between 3.0-4-5 ppm.

Total methyl content based on ^{1}NMR: mol (CH_3) / mol (CD) = 5.4

Total tosyl content based on ^{1}H-NMR: mol (tosyl) / mol (CD) = 0.3

Free tosic acid content based on CE: < 0.02 % (w/w).

Viscosity of a 1% w solution: 1.117 cPoise.

(Coumarin)$_m$-(methyl)$_n$-poly-β-CD **(6)**

4-Methyl-7-hydroxy-coumarin (60 mg, 0. 32 mmol) was added to a viscous suspension of DMF (20 mL), compound **5** (0.5 g) and Cs_2CO_3. (210 mg, 0.64 mmol). The mixture was stirred at 90-100 °C for 24 h under nitrogen. The crude was centrifuged in order to remove the undissolved Cs_2CO_3 and the supernate was evaporated till dryness under reduced pressure (T=80 °C). The residual solid was dissolved in H_2O (10 mL) and extracted with EtAc (3 times 50 mL) to remove untagged 4-methyl-7-hydroxy-coumarin. The aqueous phase was dialyzed against deionized water for 24 h. The dialysate was evaporated to dryness under reduced pressure (T= 80 °C), the residual solid was dissolved in H_2O (10 mL) and extracted with EtAc (3 times 50 mL). The aqueous phase was concentrated under reduced pressure (T= 80 °C) and dialyzed for 5 h against deionized water. Complete evaporation of the dialysate under reduced pressure (T=60 °C) yielded compound **6** as a slightly yellow glassy material (0.35 g).

In the ^{1}H-NMR spectrum (D_2O) the only peak of the dye that is detectable is the methyl group at position four at around 2.0 ppm.

Assignments based on HSQC and DEPT spectra (D_2O): δ= (proton, carbon) X, (3.39, 58.31) CD-OCH_3, (3.46, 56.92) EPI-OCH_3, (3.56, 58.88) CD-OCH_3, (3.55, 80.22) C4, (3.57, 59.03) CD-OCH_3, (3.58, 70.24) Cα-Cγ, (3.61, 62.54) Cα-Cγ (glycol type), (3.66, 70.24) C6 sub., (3.67, 78.78) C2 sub., (3.86, 60.01) C6 unsub., (3.89, 70.49) (C3,C5), (4.01, 68.72) Cβ, (5.06, 100.8) C1', (5.20, 99.08) C1.

The CD content was assumed as in β-CD polymer: 70 % (w/w) corresponds to the integral value of 77.89 protons between 3.0-4-5 ppm.

Total methyl content based on ^{1}NMR: mol (CH_3) / mol (CD) = 4.8

Total 4-methyl-7-hydroxy-coumarin content based on ^{1}H-NMR: mol (coumarin) / mol(CD)=0.12.

Free 4-methyl-7-hydroxy-coumarin content based on CE: < 0.03 % (w/w).

Residual tosic acid content based on CE: < 0.01 % (w/w).

Poly-$(N_3)_1$-β-CD **(7)**

6-monoazido-6-monodeoxy-β-CD (17.8 g, 15.3 mmol) was suspended in water (50 mL), NaOH (4.4 g, 110 mmol) was added and stirred for 10 min then epichlorohydrin (8.5 mL, 0.1 mmol) was added and the reaction mixture and heated at 70 °C for 3 h. The solution was cooled down to r.t, neutralized with HCl (5 N), dialyzed against deionized water and freeze-drying yielded compound **7** as a white powder (11.87 g).

IR (KBr) ν/cm^{-1}: 3412 (O-H), 2928 (C-H), 2106 (azido), 1652, 1456, 1410, 1366, 1330, 1159, 1084, 1036, 856, 760, 708, 580.

Assignments based on HSQC-DEPT ($(CD_3)_2SO$): δ= (proton, carbon) X, (3.31, 73.42) Cα-Cγ, (3.31, 81.90) C4, (3.32, 57.80) C6-N_3, (3.32, 63.16) Cα-Cγ (glycol type), (3.38, 72.89) Cα-Cγ,

(3.45, 82.44) C2 sub., (3.51, 51.37) C6-N_3, (3.56, 71.91) C3-C5, (3.57, 51.37) C6-N_3, (3.61, 62.39) C6 unsub., (4.82, 101.91) C1, (4.99, 101.23) C1'.

CD content based on ^{1}NMR: 66 % (w/w).

N_3 content based on ^{1}H-NMR: 2.4 % (w/w).

Molecular weight based on SLS: 58 ±3 kDa.

Viscosity of a 1% w solution: 1.075 cPoise.

Poly-(NH_2)$_1$-β-CD **(8)**

Compound **7** (1 g) was dissolved in H_2O (30 mL), MeOH (5 mL) was added and the solution was heated till 50 °C. First Pd/C suspension (0.3 g, 10% Pd content in 3 mL H_2O), then hydrazine hydrate (1 mL, 1 g, 0.02 mmol) was added and heated to reflux. After 12 h stirring under reflux, the reaction mixture was cooled to about 40 °C, most of the catalyst was filtered off and the mother liquor was centrifuged in order to remove the remaining Pd/C. The supernate was dialyzed against deionized water for 24 h and freeze-drying yielded compound **8** as a white powder (0.67 g). The material was stored under inert atmosphere till the utilization.

Assignments based on HSQC-DEPT ((CD_3)$_2$SO): δ= (proton, carbon) X, (3.61, 80.63) C4, (3.65, 60.56) Cα-Cγ (glycol type), (3.62, 72.38) Cα-Cγ, (3.88, 60.14) C6 unsub., (3.90, 70.38) C3-C5, (3.95, 72.96) Cβ, (5.08, 100.95) C1.

IR (KBr) ν/cm^{-1}: disappearance of the azide band (2103), 3377 (O-H), 2926 (C-H), 1652, 1429, 1363, 1088, 1045, 946, 694, 652, 549.

CD content based based on ^{1}NMR: 67 % (w/w).

Molecular weight based on SLS: 62 ± 5 kDa.

(NBF-NH)$_n$-poly-(NH$_2$)$_1$-β-CD **(9)**

NBF-Cl (0.1 g, 0.5 mmol) was dissolved in CH_3CN (2 mL) and added to an aqueous solution (10 mL) of compound **8** (1 g). The reaction mixture was stirred at 60-70 °C for 5 h, the solvents were evaporated completely under reduced pressure (T=60 °C) and the crude was dissolved in H_2O (20 mL). The dark brown solution was extracted twice with DCM (100 mL), then the aqueous phase was dialyzed against deionized water for 24 h and complete evaporation of the solvent yielded compound **9** as a dark brown powder (0.7 g). The material was stored under inert atmosphere.

In the ^{1}H-NMR spectrum (D_2O) the aromatic peaks of the NBF groups appear around 6.5 ppm and around 8.5 ppm as broad signals.

In the ^{13}C-NMR spectrum (D_2O) the peak of the C6-NH_2 is visible at 40.98 ppm.

CD content based on ^{1}NMR: 65 % (w/w).

Total NBF content based on ^{1}H-NMR: mol (NBF) / mol (CD) = 0.1

Free NBF-Cl content based on TLC: <0.1 % (w/v).

Molecular weight based on SLS: 52 ± 4 kDa.

(6-Deoxy)$_n$-6-[(5/6)-fluoresceinylthioureido]$_n$- poly-β-CD **(10)**

6-monodeoxy-6-[(5/6)-fluoresceinylthioureido]-β-CD (0.1 g, 0.066 mmol) and β-CD (10 g, 8.81 mmol) were suspended in water (30 mL), NaOH (2.5 g, 62.5 mmol) was added and stirred for 10 min then epichlorohydrin (4.8 mL, 60 mmol) was added and the reaction mixture was heated at 70 °C for 3 h. The solution was cooled down to r.t, neutralized with HCl (5 N), dialyzed against deionized water for 48 h and freeze-drying yielded compound **10** as a yellow powder (8.23 g).

IR (KBr) ν/cm^{-1}: 3413 (O-H), 2926 (C-H), 1646, 1592, 1466, 1328, 1151, 1082, 1041, 853, 761.

In ^{1}H-NMR spectrum (D_2O) the peaks of the fluoresceinyl moiety appear at 6.74 ppm and at 7.28 ppm as broad signals.

Assignments based on HSQC-DEPT ($(CD_3)_2SO$): δ= (proton, carbon) X, (3.59, 72.21) Cα-Cγ, (3.60, 80.47) C4, (3.62, 62.66) Cα-Cγ (glycol type), , (3.76, 77.97) C2 sub., (3.80, 69.50) C6 sub., (3.88, 60.27) C6 unsub., (3.89, 70.37) C3, (3.94, 72.54) C5, (4.02, 68.96) Cβ, (5.22, 99.14) C1.

CD content based on ^{1}NMR: 69 % (w/w).

Total fluorescein content based on ^{1}H-NMR: mol (Fluorescein) / mol (CD) = 0.02.

Free fluorescein content based on CE measurement: < 0.01 % (w/w).

Molecular weight based on SLS: 39 ± 3 kDa.

Supporting Information

See electronic supplementary information for IR, ^{1}H, ^{13}C or HSQC-DEPT spectra of the synthesized compounds.

References

[1] Renard, E.; Deratani, A.; Volet, G.; Sebille, B., *Eur. Polym. J.*, **1997**, *33*, 1, 49-57.

[2] Puskás, I.; Szemjonova, A.; Fenyvesi, É.; Malanga, M.; Szente, L., *Carbohydrate Polymers*, **2013**, *94*, 124-128.

<u>*(6-azido-6-deoxy)$_n$-poly-β-CD*</u> (**1**)
IR spectrum

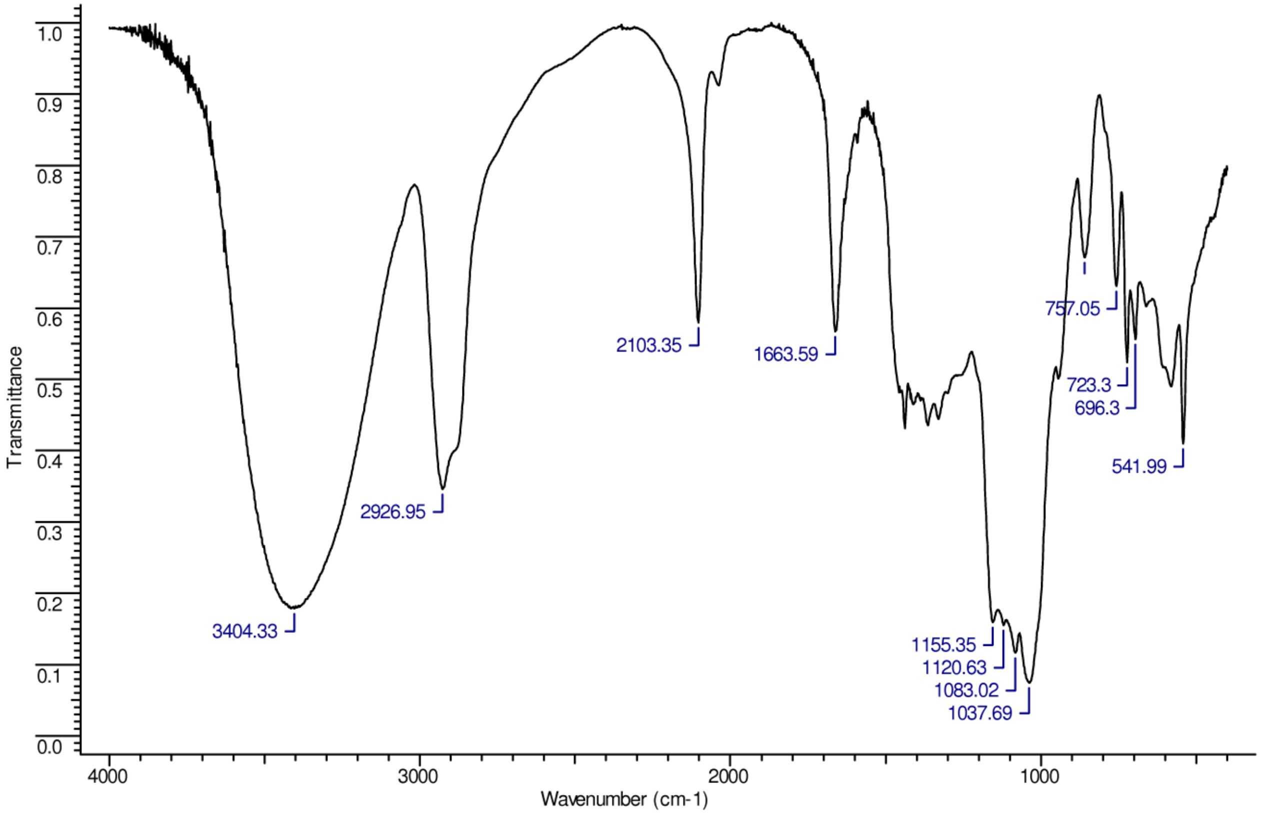

Figure S1

(6-azido-6-deoxy)$_n$-poly-β-CD **(1)**

Calibration curve for quantification of azido moieties

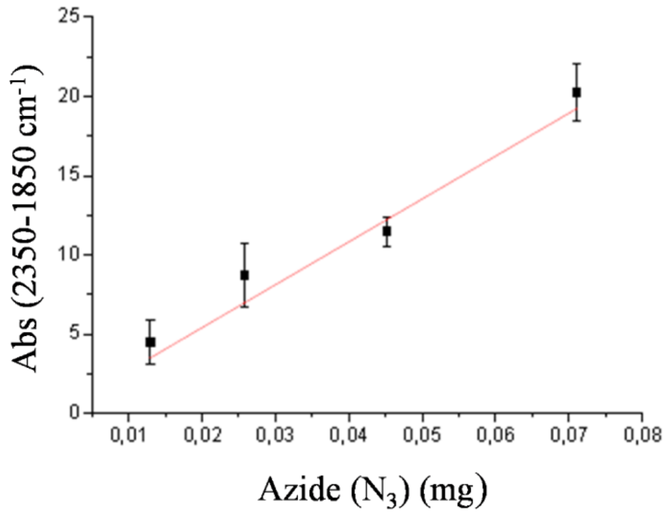

Figure S2

(6-azido-6-deoxy)$_n$-poly-β-CD **(1)**

^{1}H-NMR

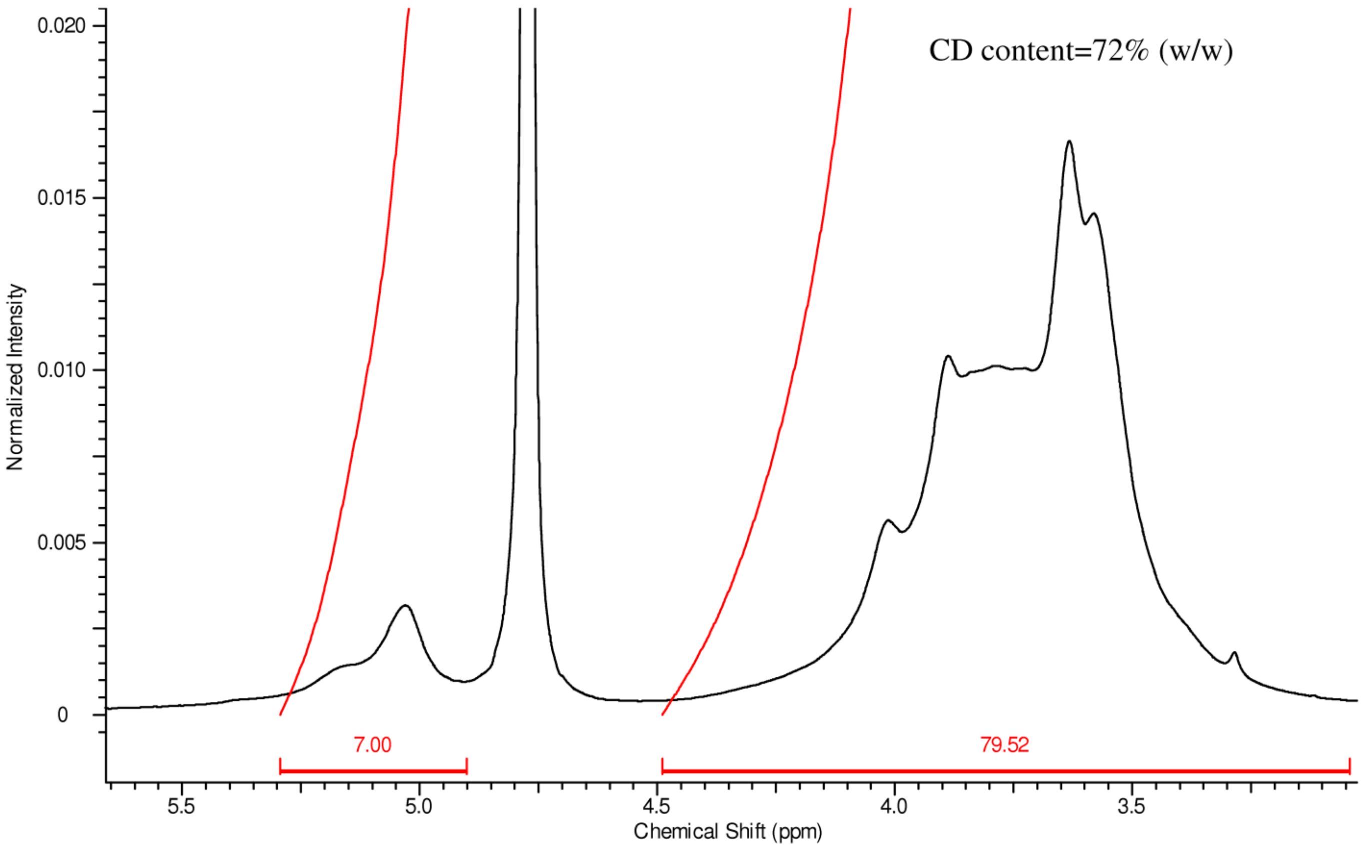

Figure S3

(6-azido-6-deoxy)$_n$-poly-β-CD **(1)**
HSQC-DEPT

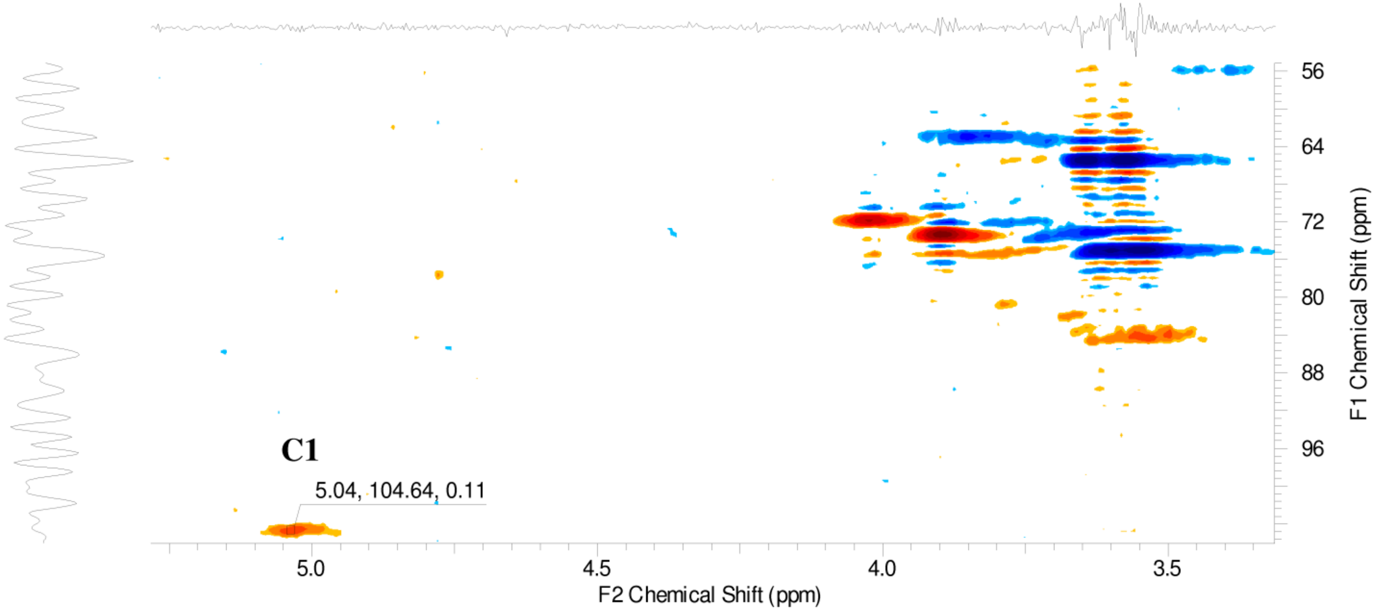

Figure S4

6-monoazido-6-monodeoxy-β-CD

HSQC-DEPT

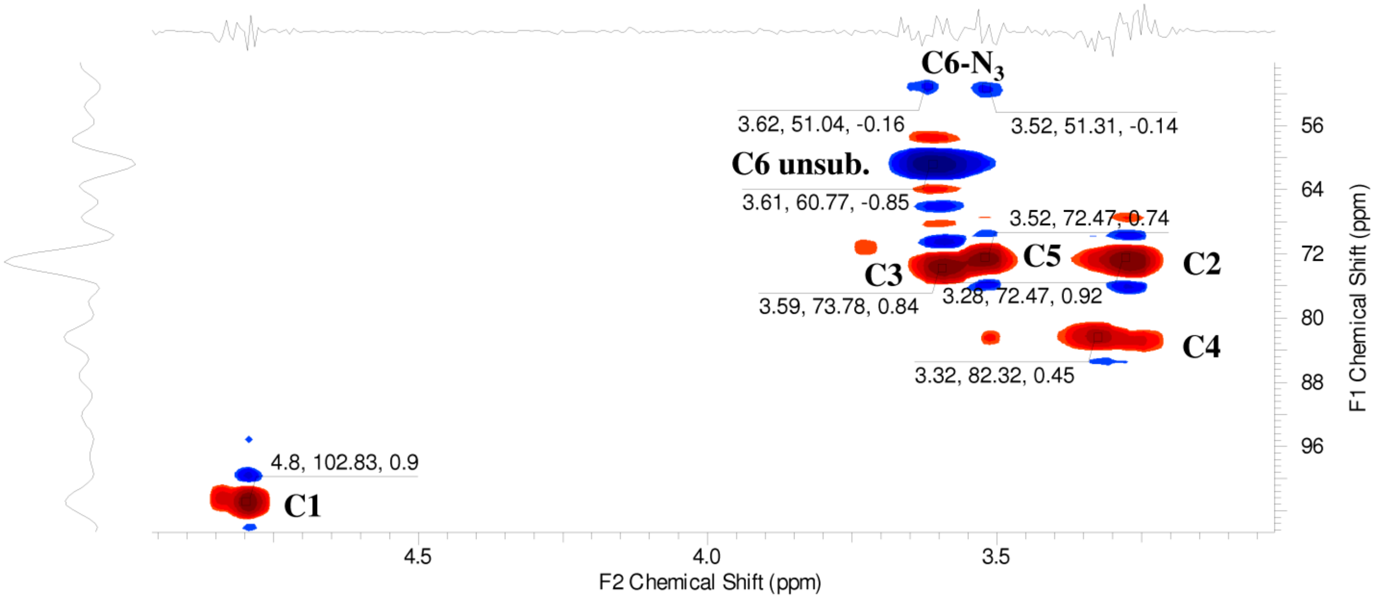

Figure S5

(6-amino-6-deoxy)$_n$-poly-β-CD **(2)**

IR-Spectra

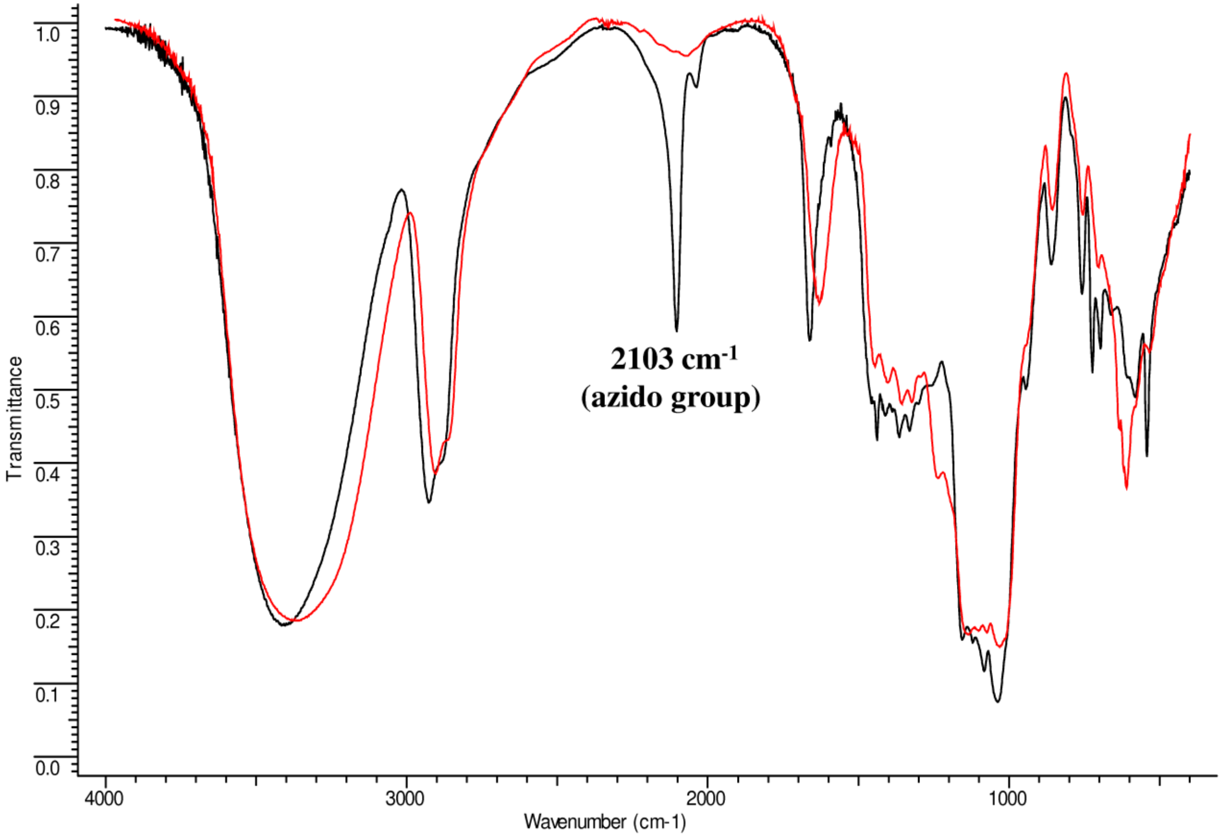

Comparison between the IR spectra of compounds **1**, black line and **2,** red line, showing the disappearance of the azido peak

Figure S6

(6-amino-6-deoxy)$_n$-poly-β-CD **(2)**

^{1}H-NMR

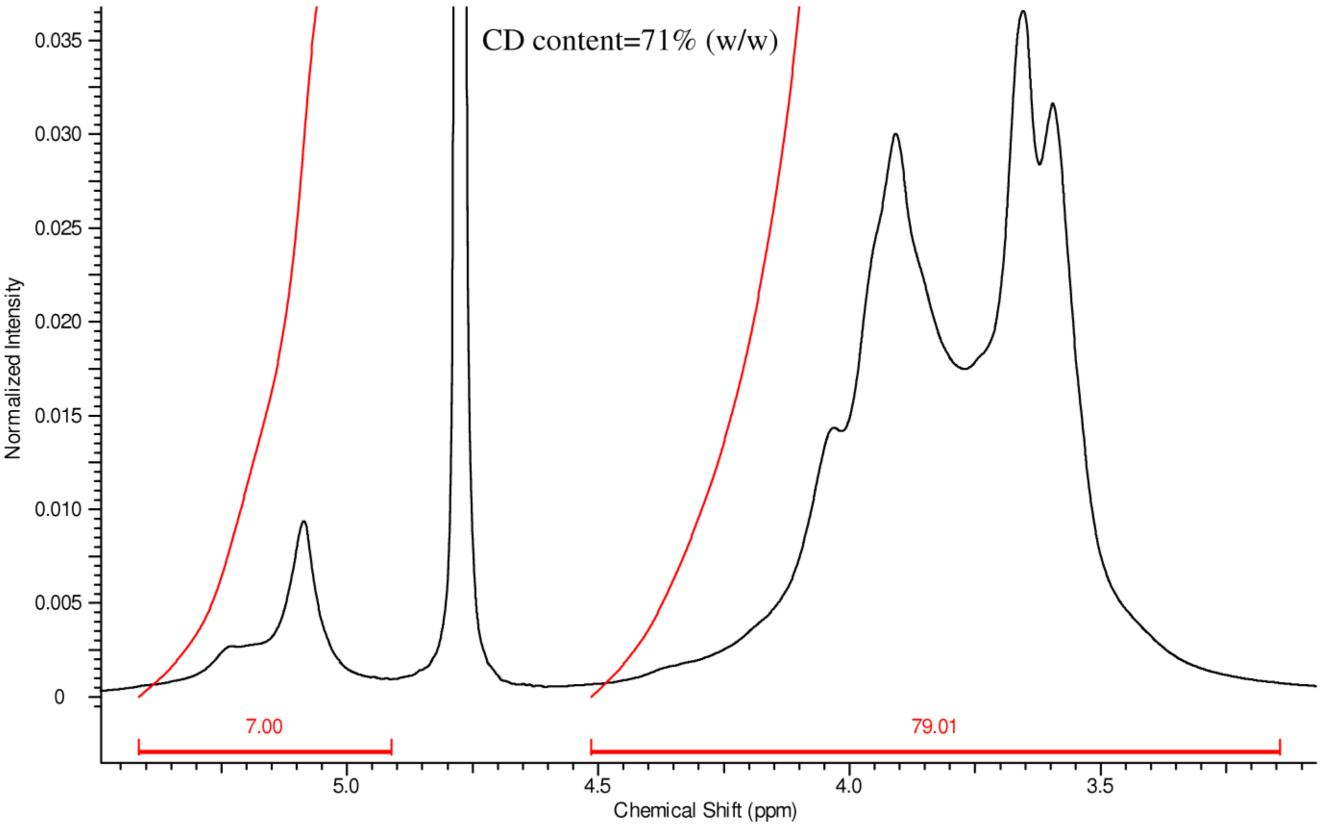

Figure S7

(6-amino-6-deoxy)$_n$-poly-β-CD (**2**)

HSQC-DEPT

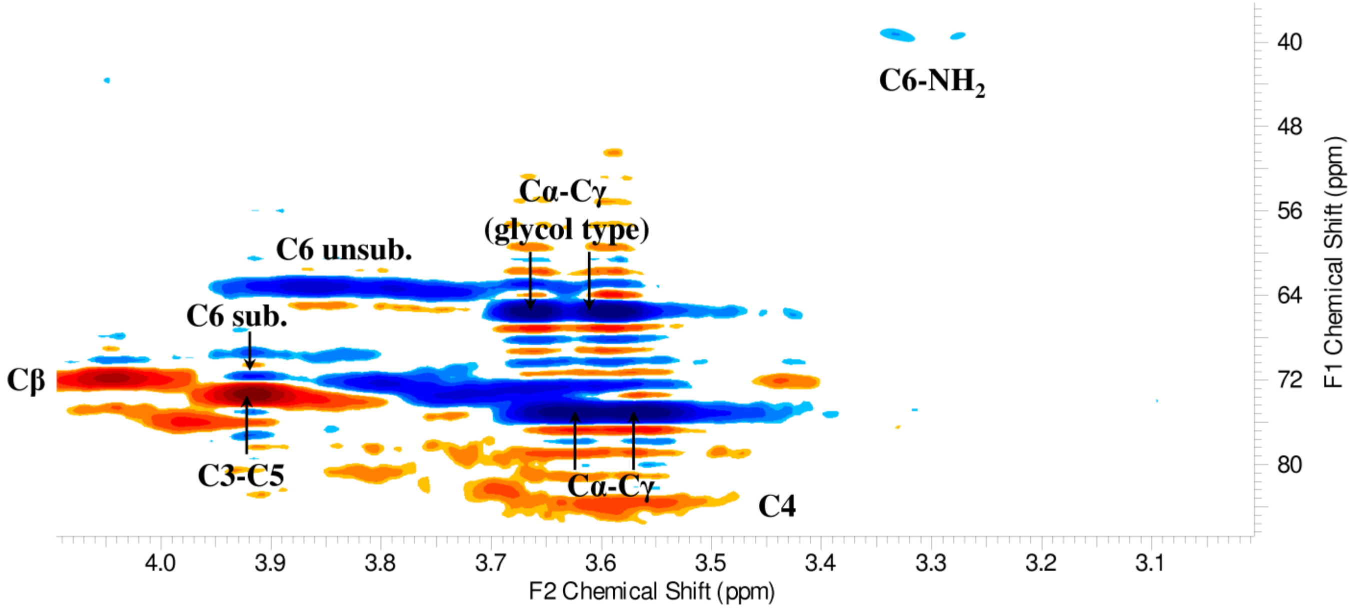

Figure S8

Per-6-amino-β-CD
HSQC-DEPT

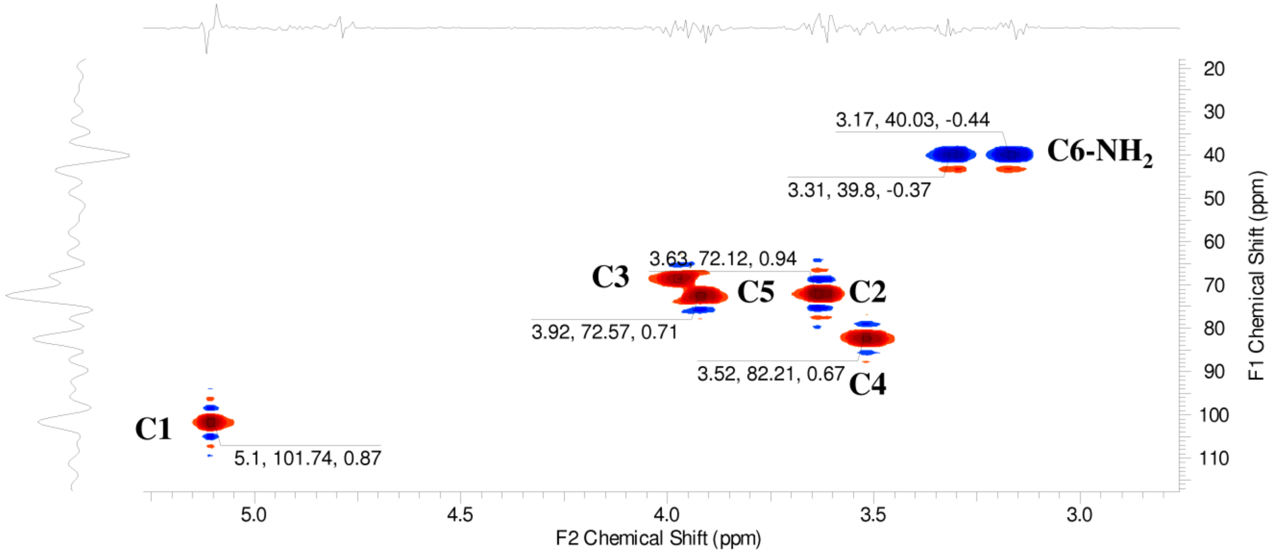

Figure S9

(6-amino-6-deoxy)$_n$-6-[(5/6)-rhodaminylthioureido]$_m$- poly-β-CD **(3)**

IR Spectrum

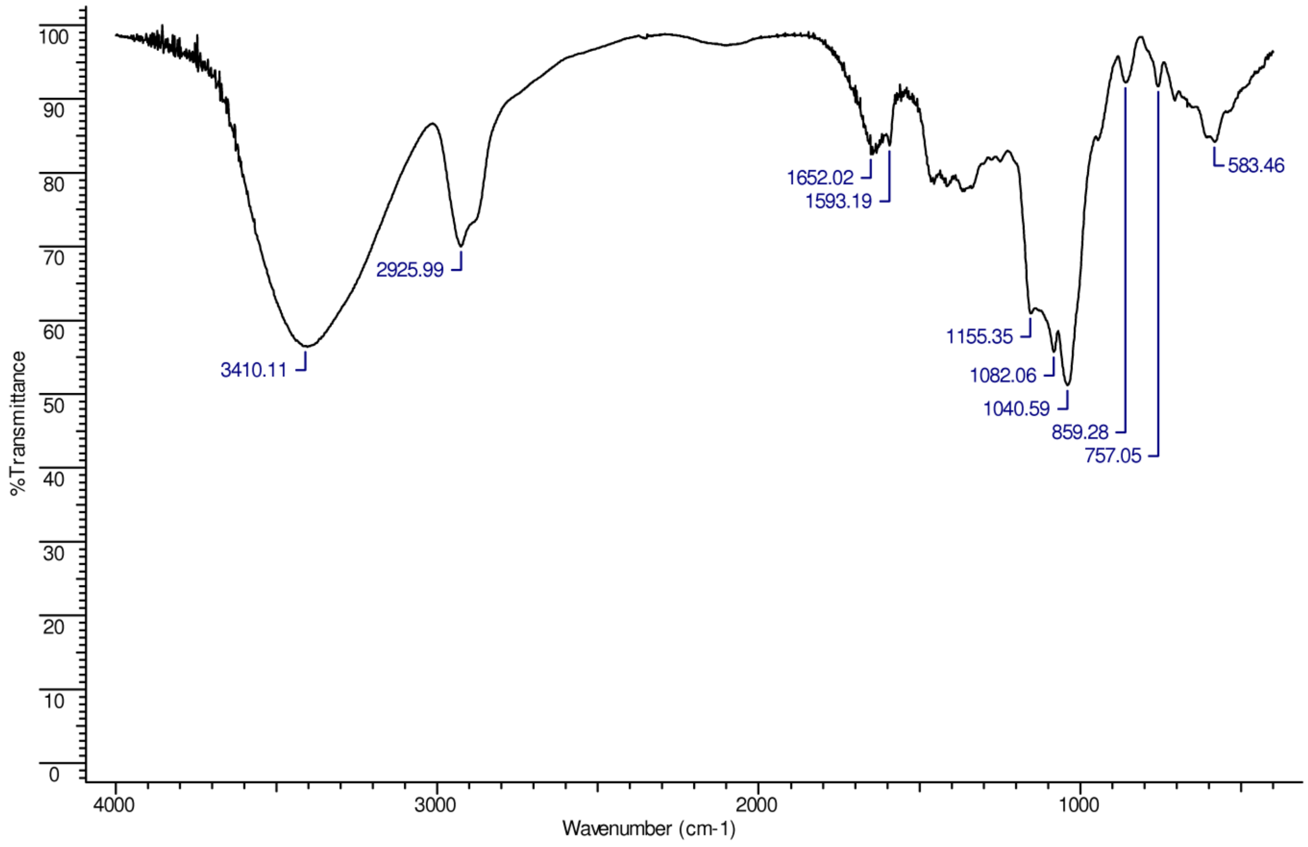

Figure S10

(6-amino-6-deoxy)$_n$-6-[(5/6)-rhodaminylthioureido]$_m$- poly-β-CD **(3)**

^{1}H-NMR

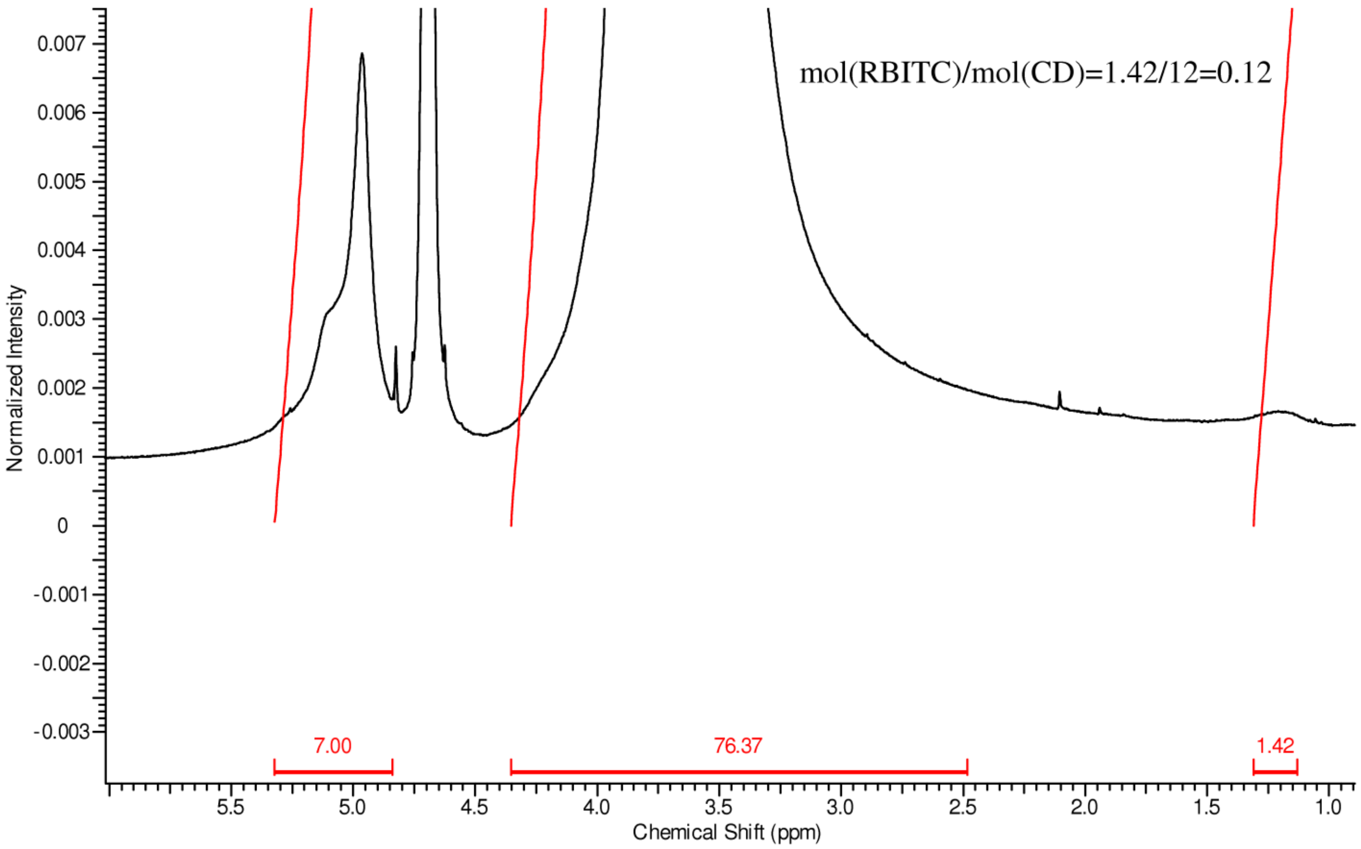

Figure S11

(Methyl)$_n$-poly-β-CD **(4)**
^{1}H-NMR

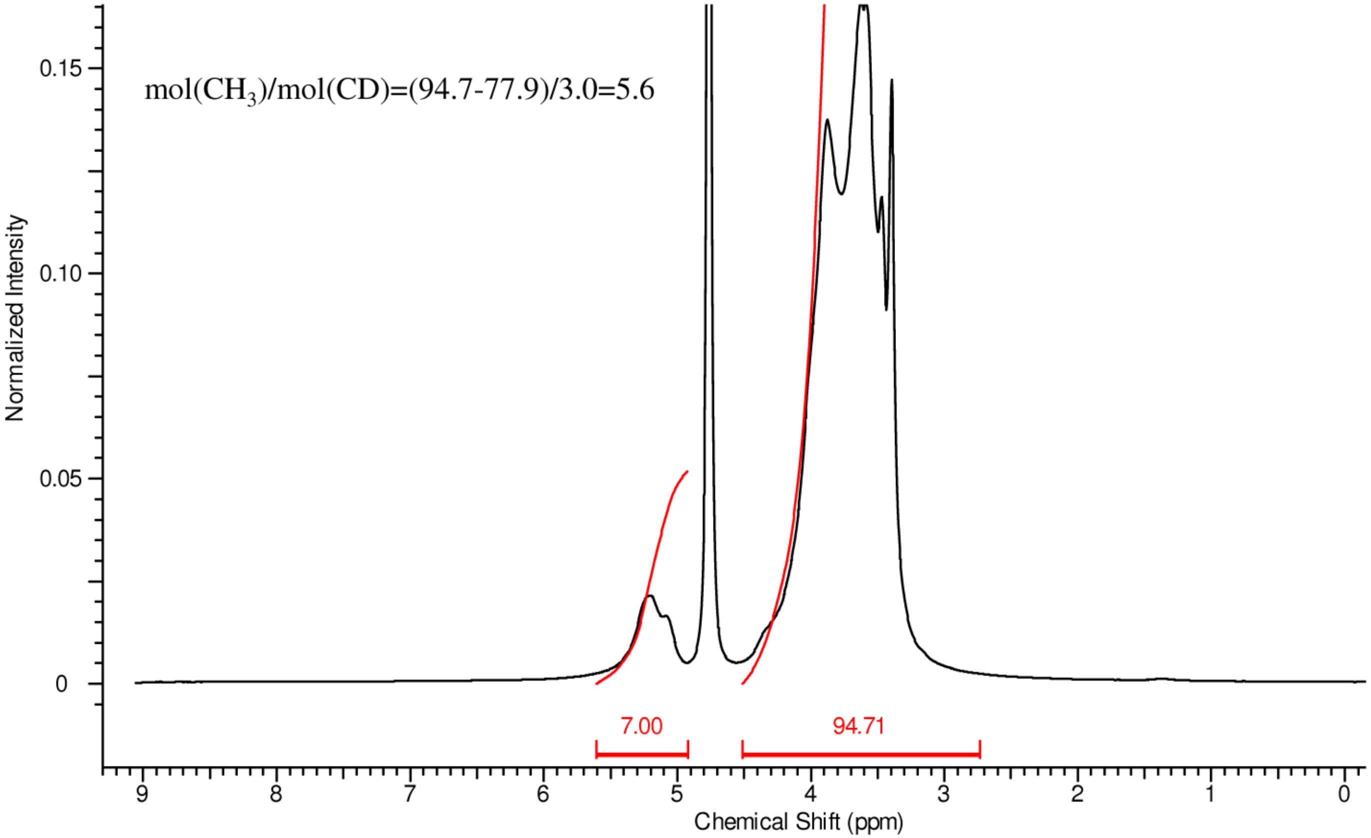

Figure S12

(Methyl)$_n$-poly-β-CD **(4)**
DEPT

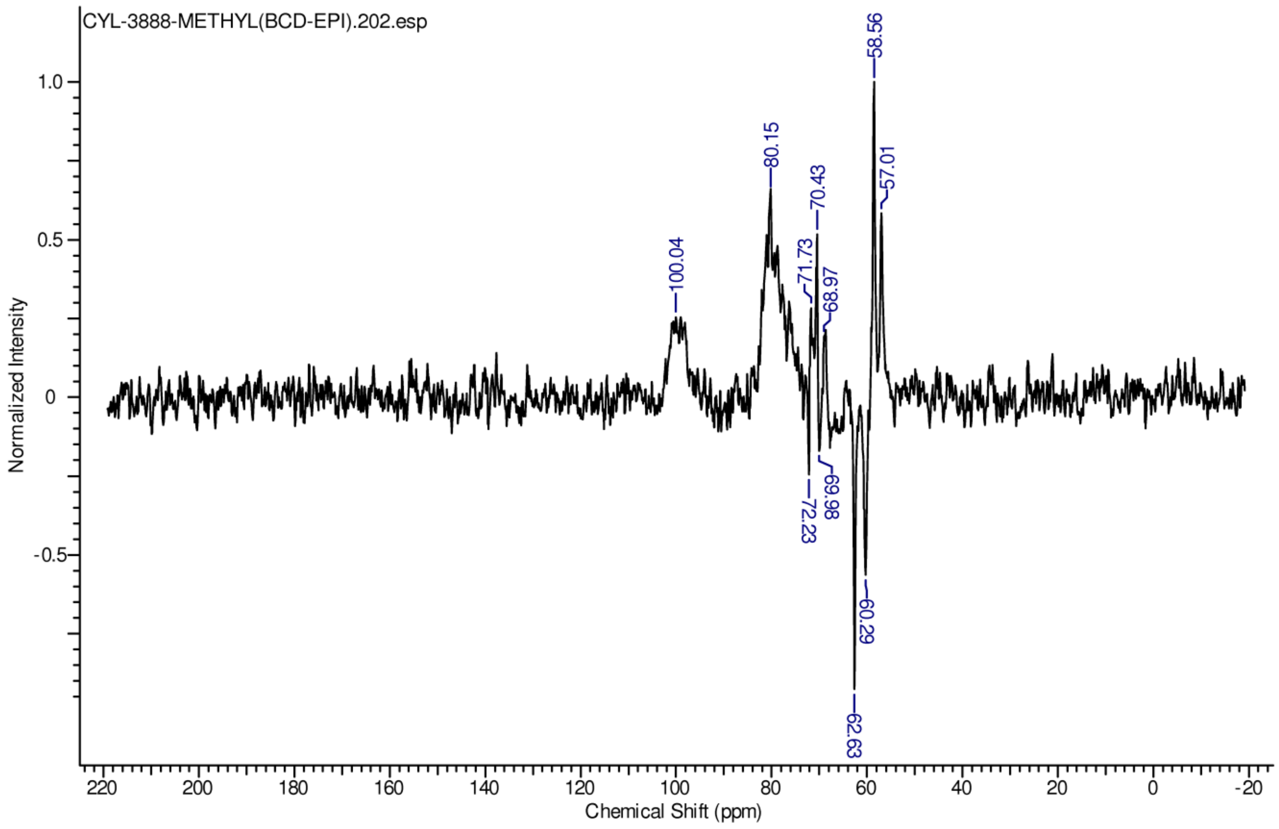

Figure S13

Methylated β-CD
HSQC-DEPT

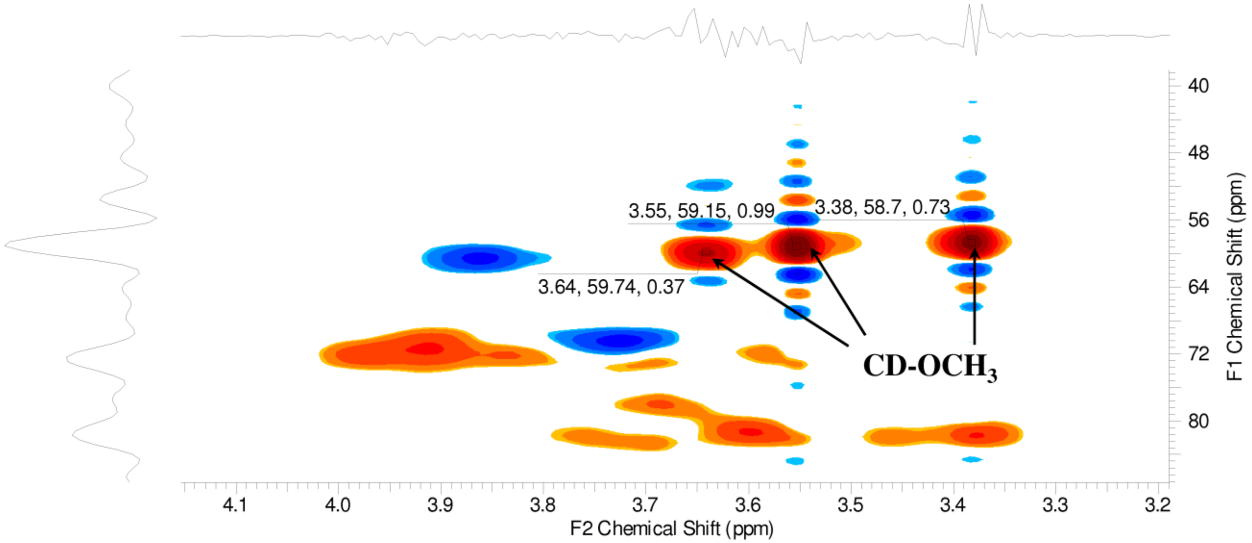

Figure S14

(Methyl)$_n$-β-CD

MS

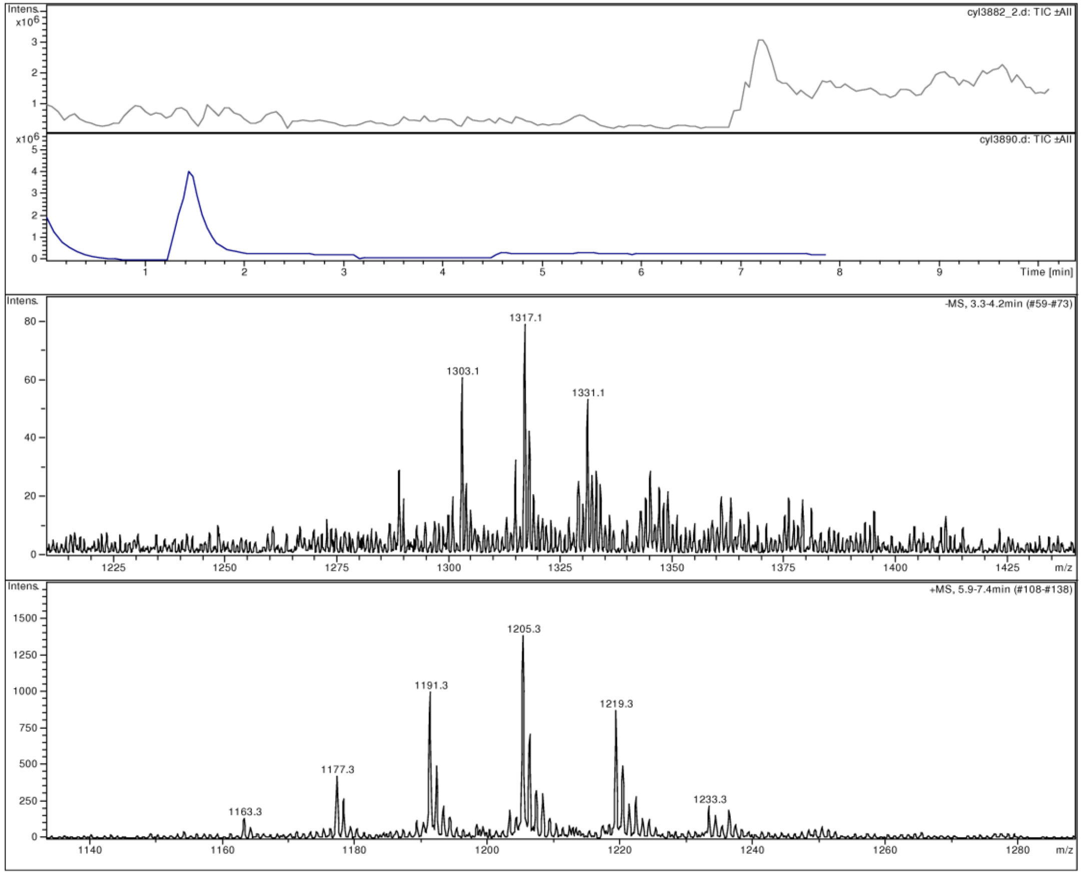

Figure S15

<u>*(Tosyl)$_m$-(methyl)$_n$-poly-β-CD*</u> **(5)**

^{1}H-NMR

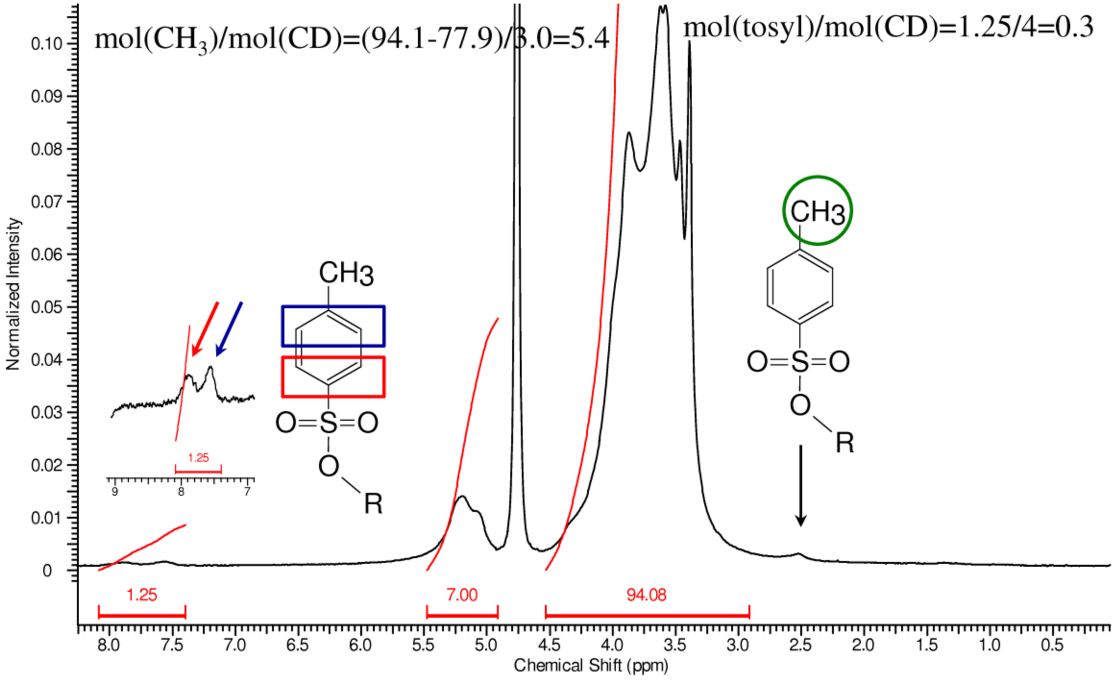

Figure S16

(Tosyl)$_m$-(methyl)$_n$-poly-β-CD **(5)**
HSQC-DEPT

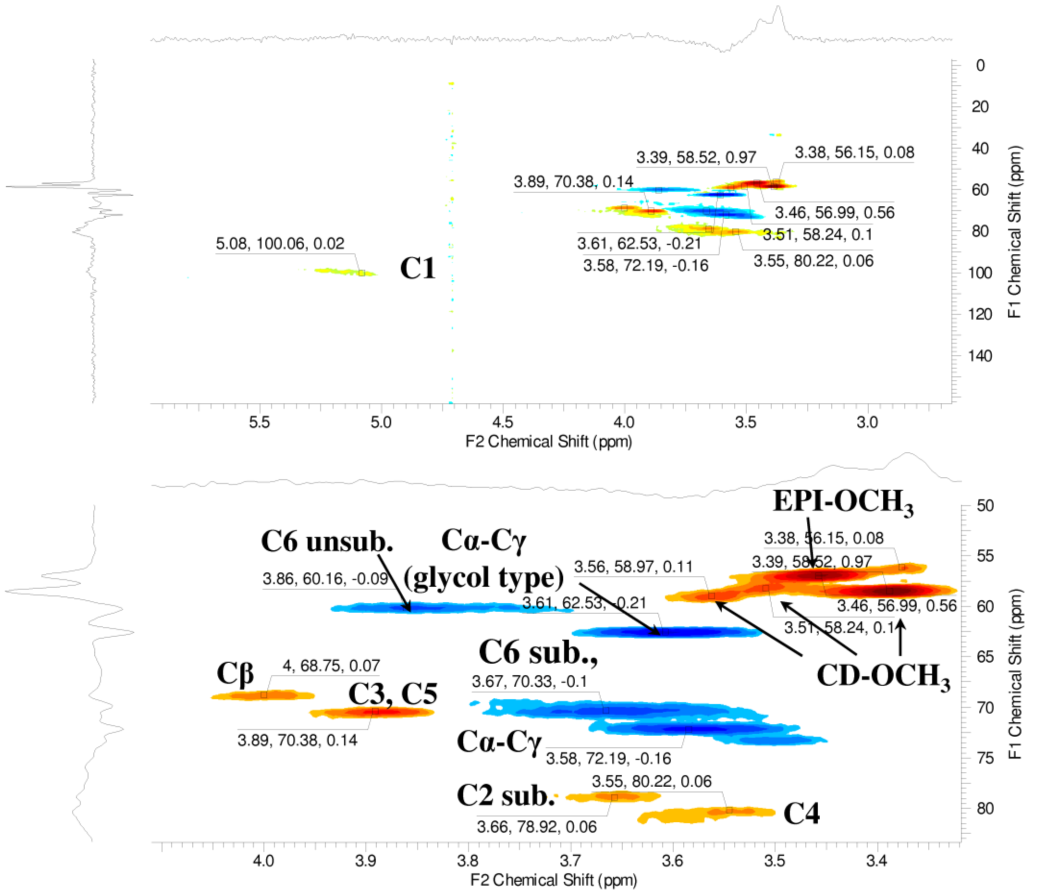

Figure S17

6-monotosyl-β-CD

HSQC-DEPT

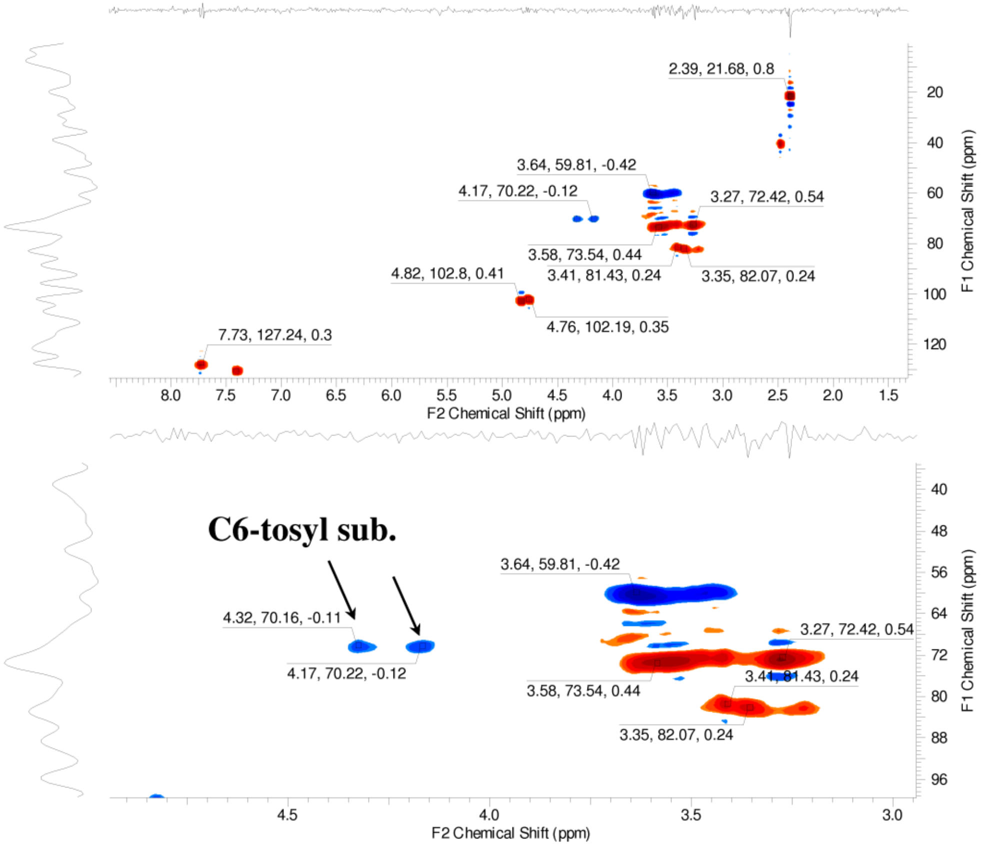

Figure S18

<u>*(Coumarin$_m$)-(methyl)$_n$-poly-β-CD*</u> **(6)**

^{1}H-NMR

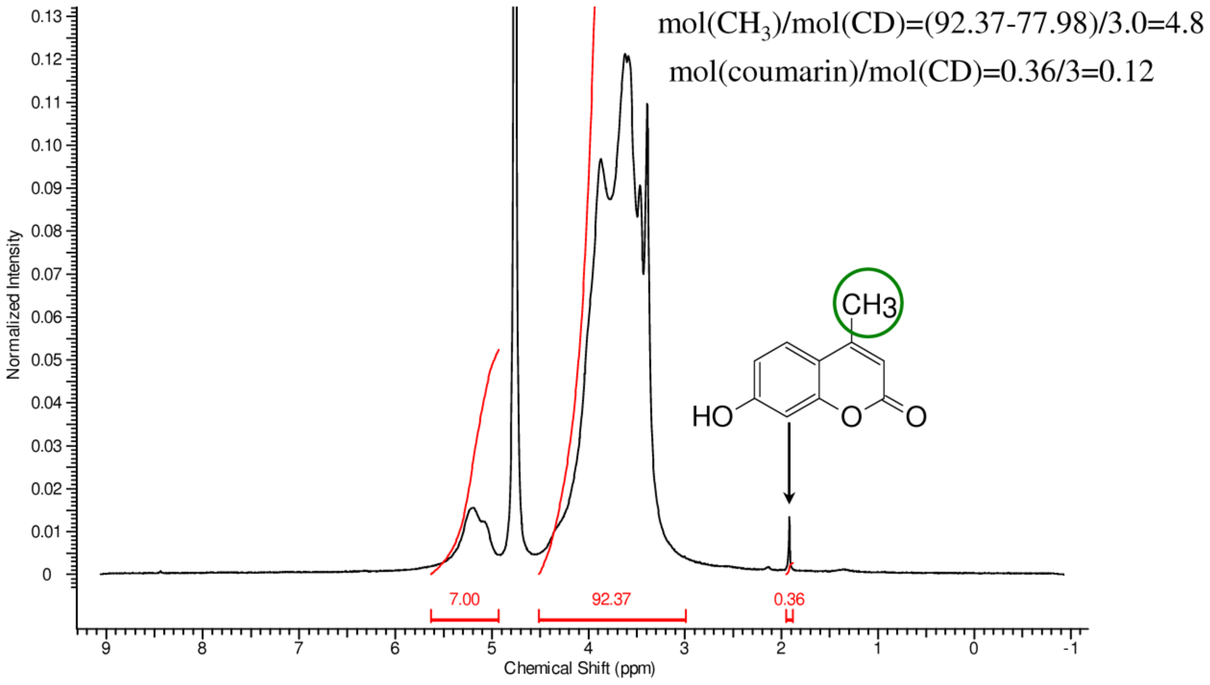

Figure S19

(Coumarin$_m$)-(methyl)$_n$-poly-β-CD **(6)**
HSQC-DEPT

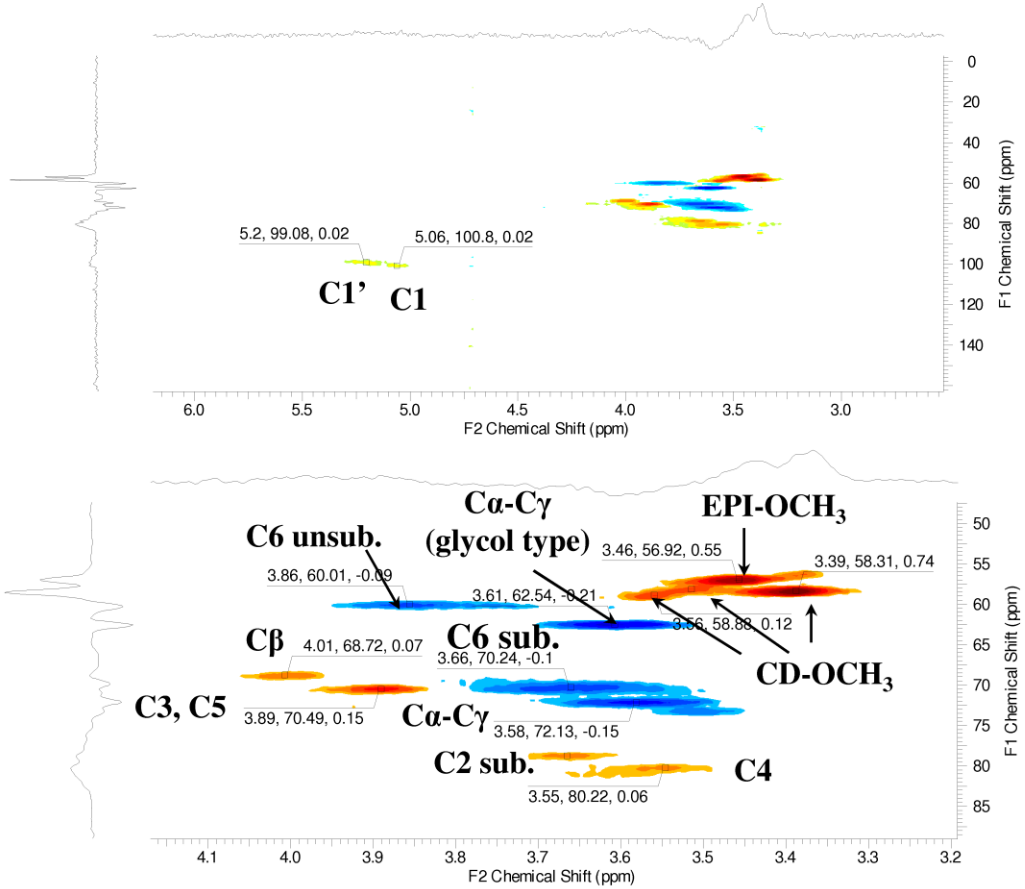

Figure S20

<u>*Poly-(N$_3$)$_1$-β-CD*</u> (**7**)

IR Spectrum

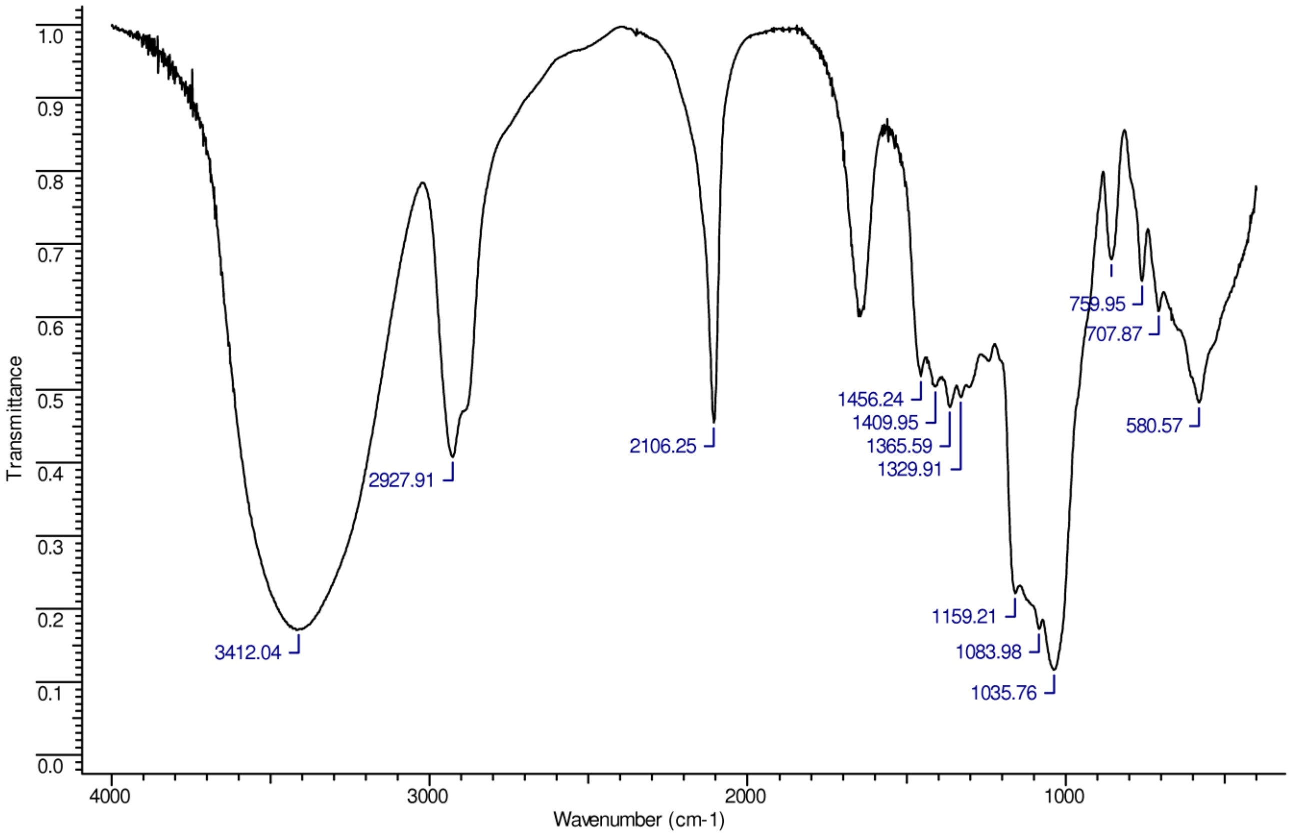

Figure S21

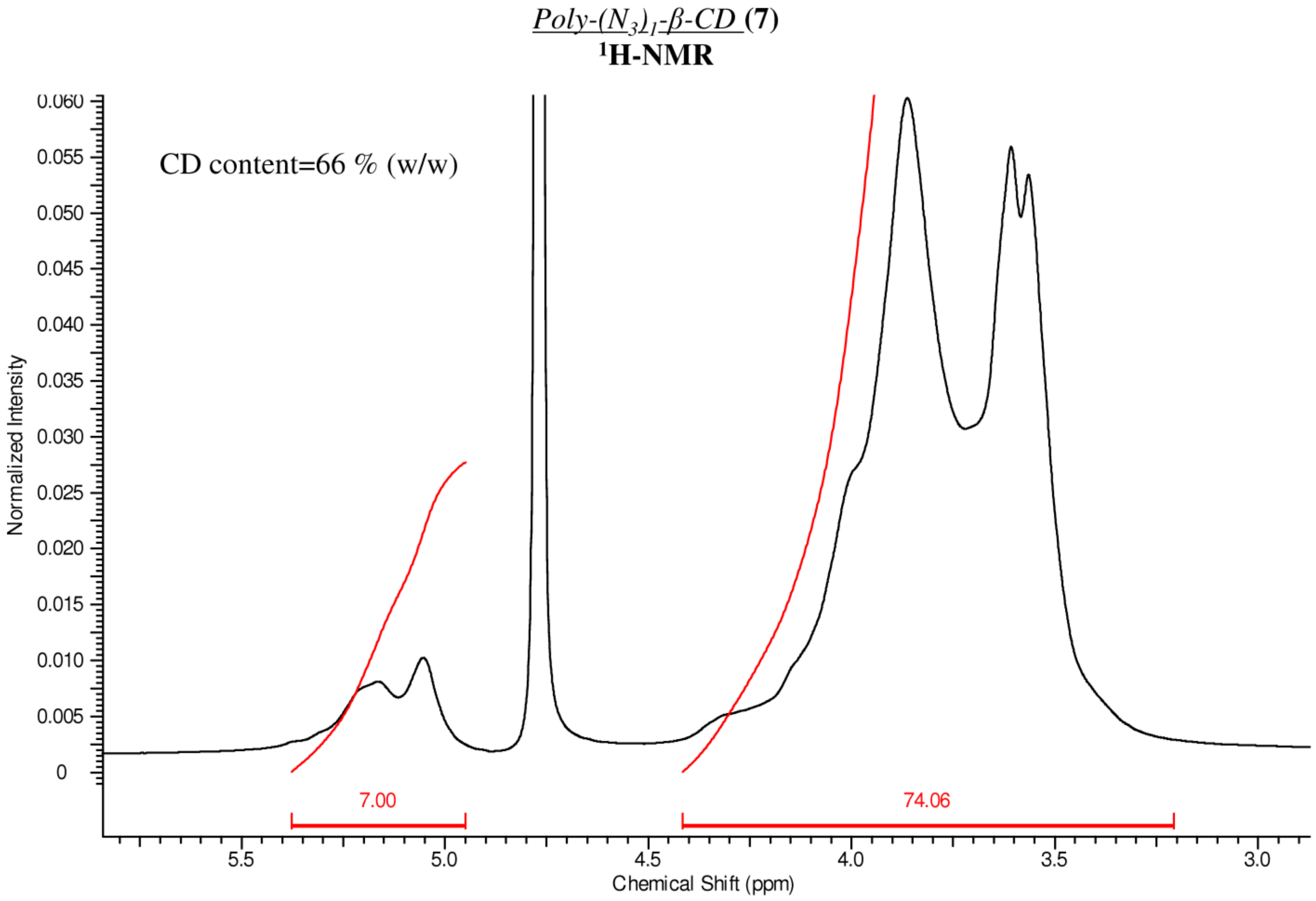

Poly-(N3)1-β-CD (7)
1H-NMR
CD content=66 % (w/w)
Normalized Intensity
0.060
0.055
0.050
0.045
0.040
0.035
0.030
0.025
0.020
0.015
0.010
0.005
0
7.00
74.06
5.5
5.0
4.5
4.0
3.5
3.0
Chemical Shift (ppm)

Figure S22

Poly-(N_3)$_1$-β-CD (7)
HSQC-DEPT

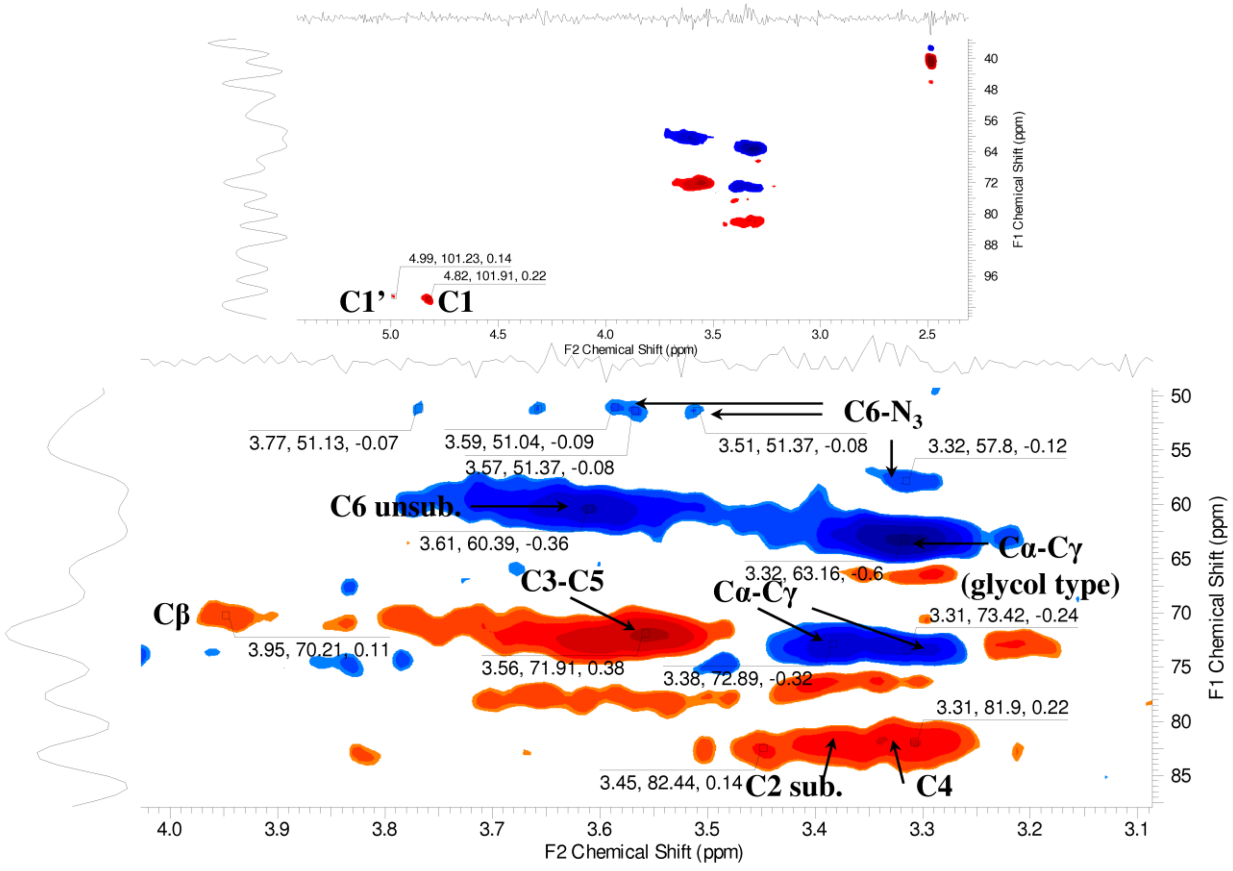

Figure S23

Poly-$(N_3)_1$-β-CD (**7**)

^{13}C-NMR

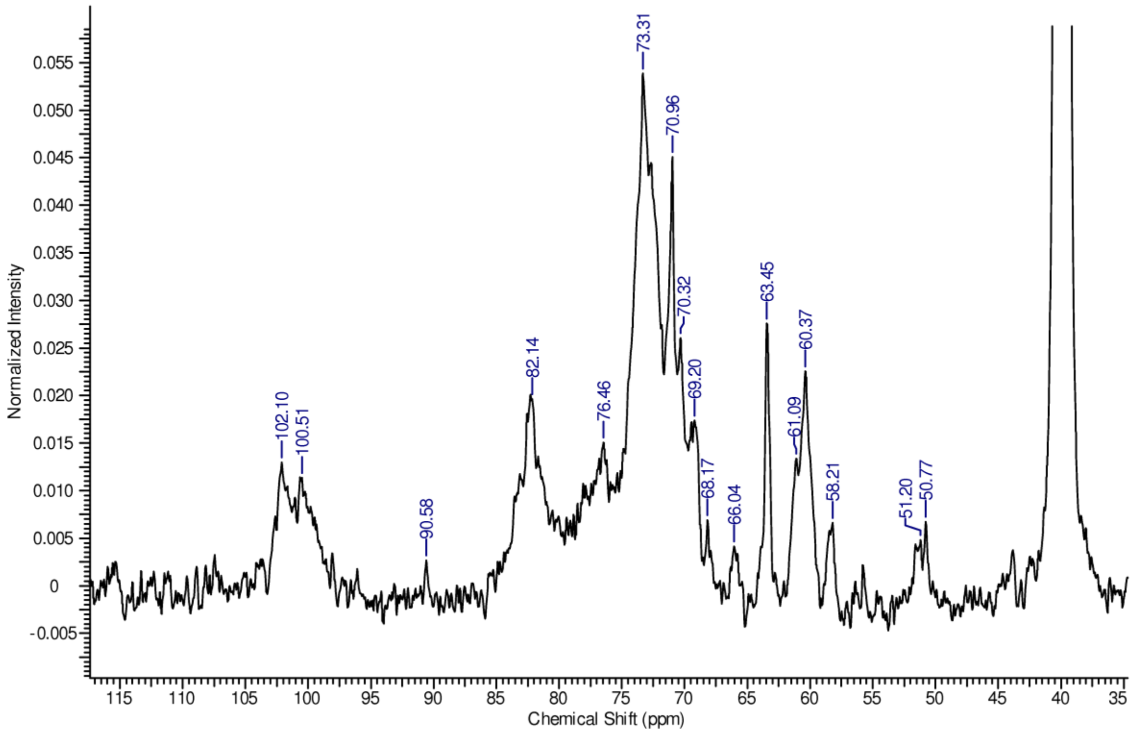

Figure S24

Poly-(NH_2)$_1$-β-CD **(8)**

IR Spectrum

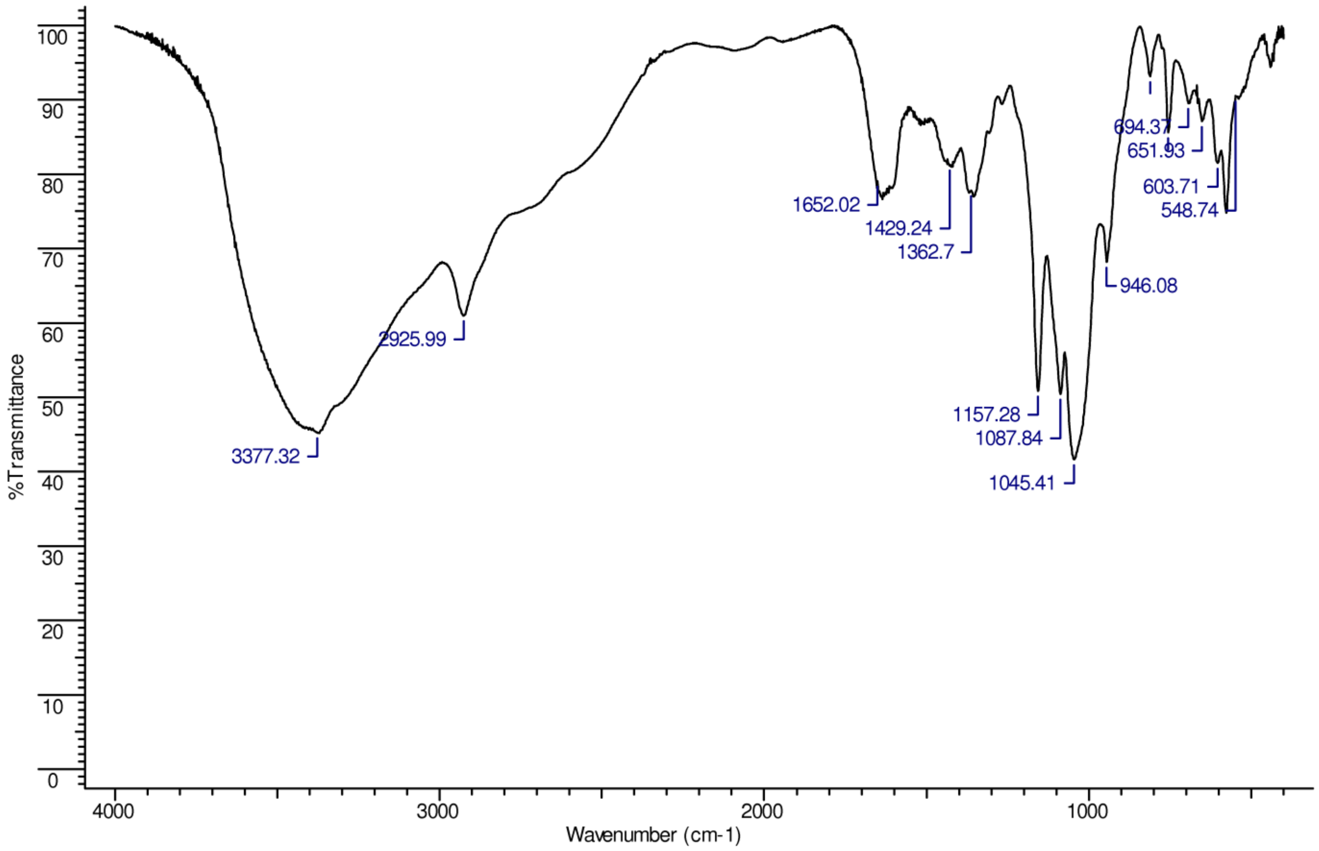

Figure S25

Poly-$(NH_2)_1$-β-CD (**8**)
^{1}H-NMR

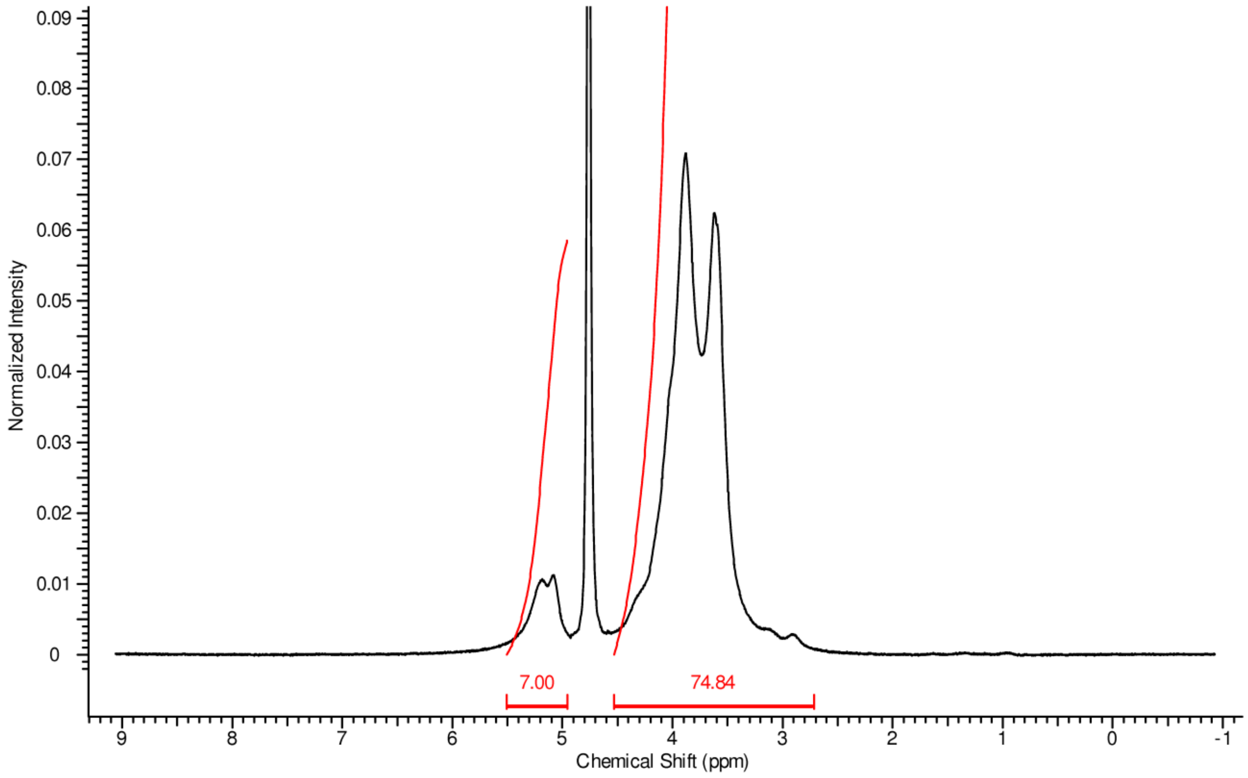

Figure S26

Poly-(NH$_2$)$_1$-β-CD **(8)**

HSQC-DEPT

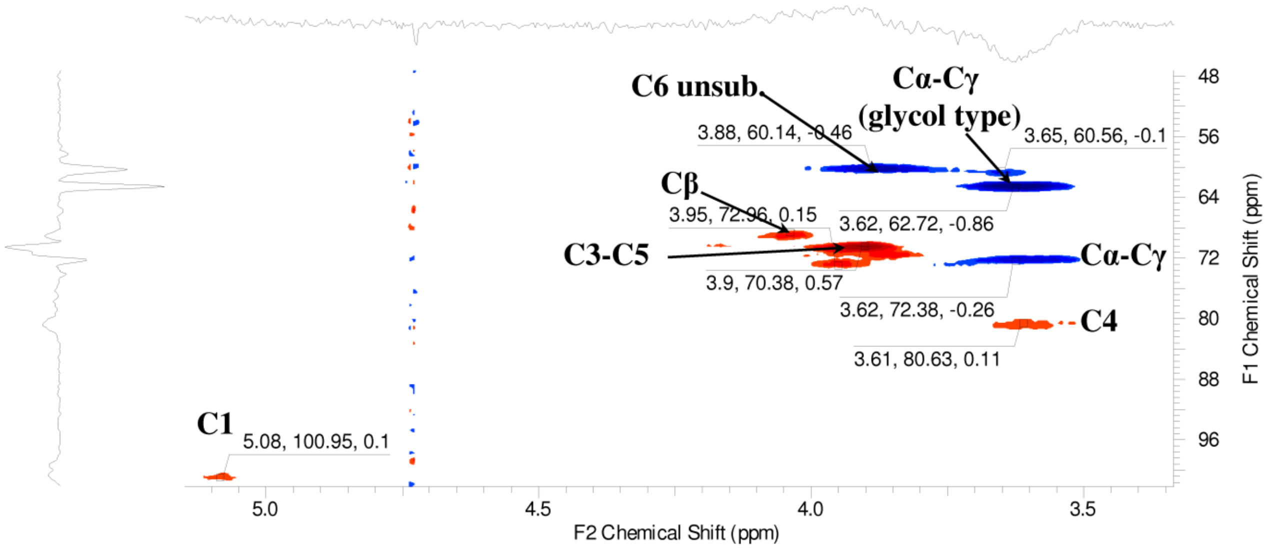

Figure S27

(NBF-NH)$_n$-Poly-(NH$_2$)$_1$-β-CD **(9)**
^{1}H-NMR

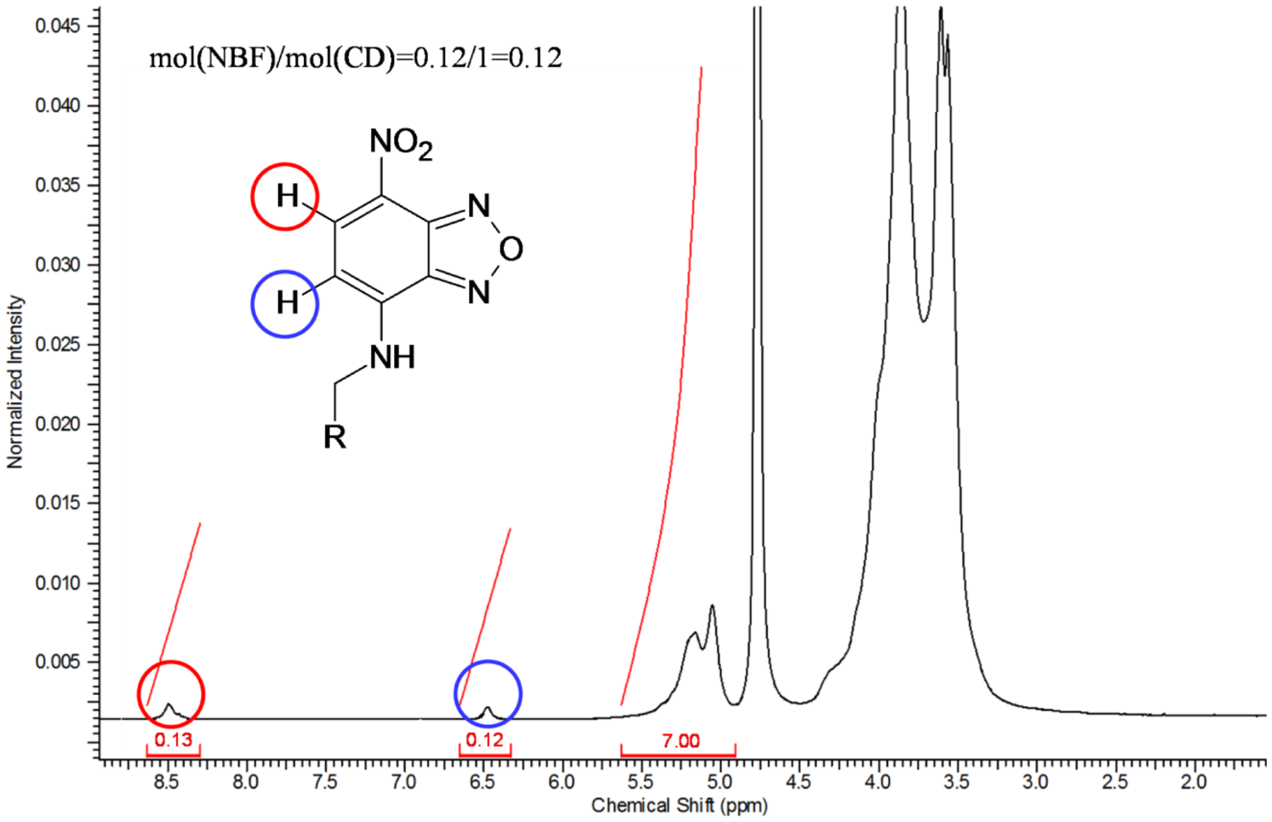

Figure S28

(NBF-NH)$_n$-Poly-(NH$_2$)$_1$-β-CD **(9)**
^{13}C-NMR

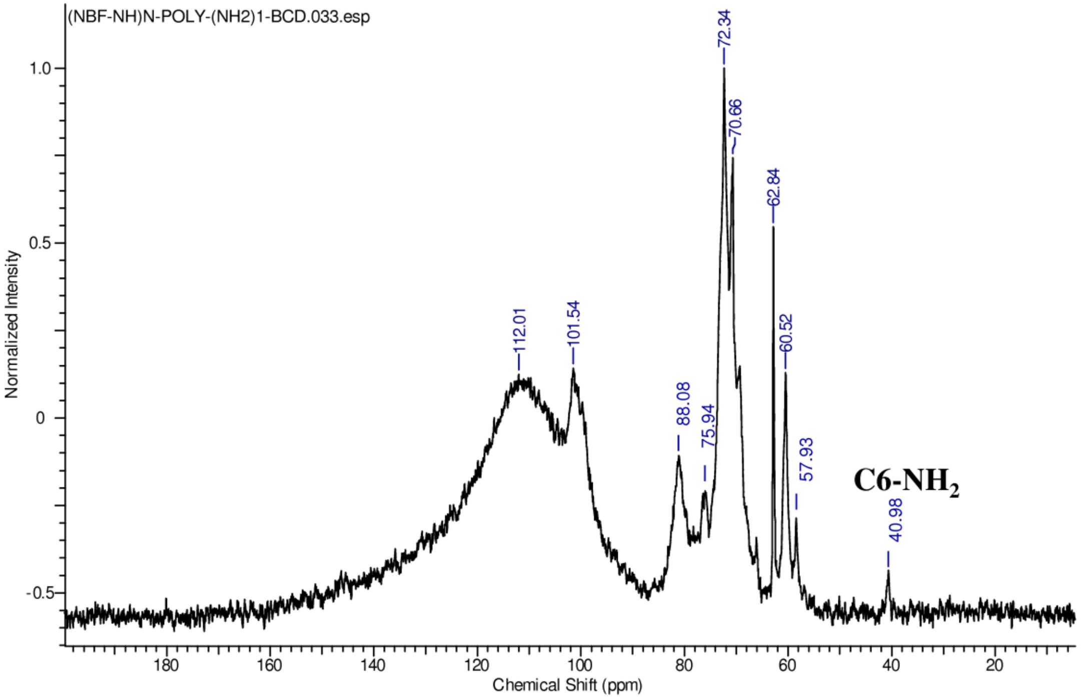

Figure S29

(NBF-NH)$_n$-Poly-(NH$_2$)$_1$-β-CD **(9)**
Free NBF-Cl by TLC

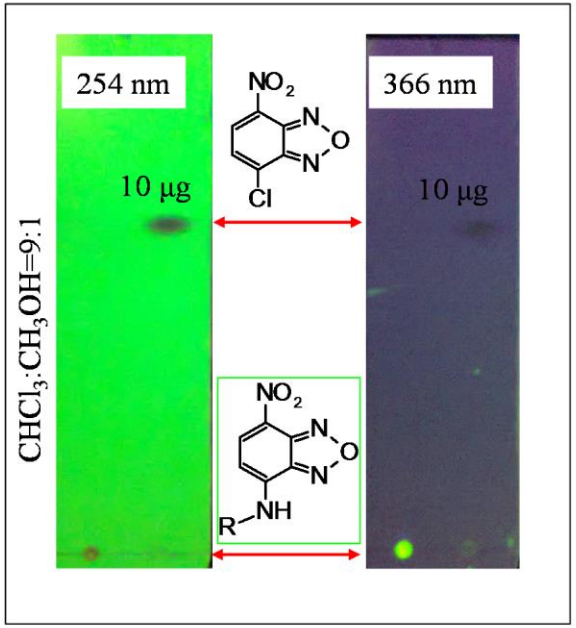

Figure S30

(6-amino-6-deoxy)$_n$-6-[(5/6)-fluoresceinylthioureido]$_m$- poly-β-CD **(10)**

IR Spectrum

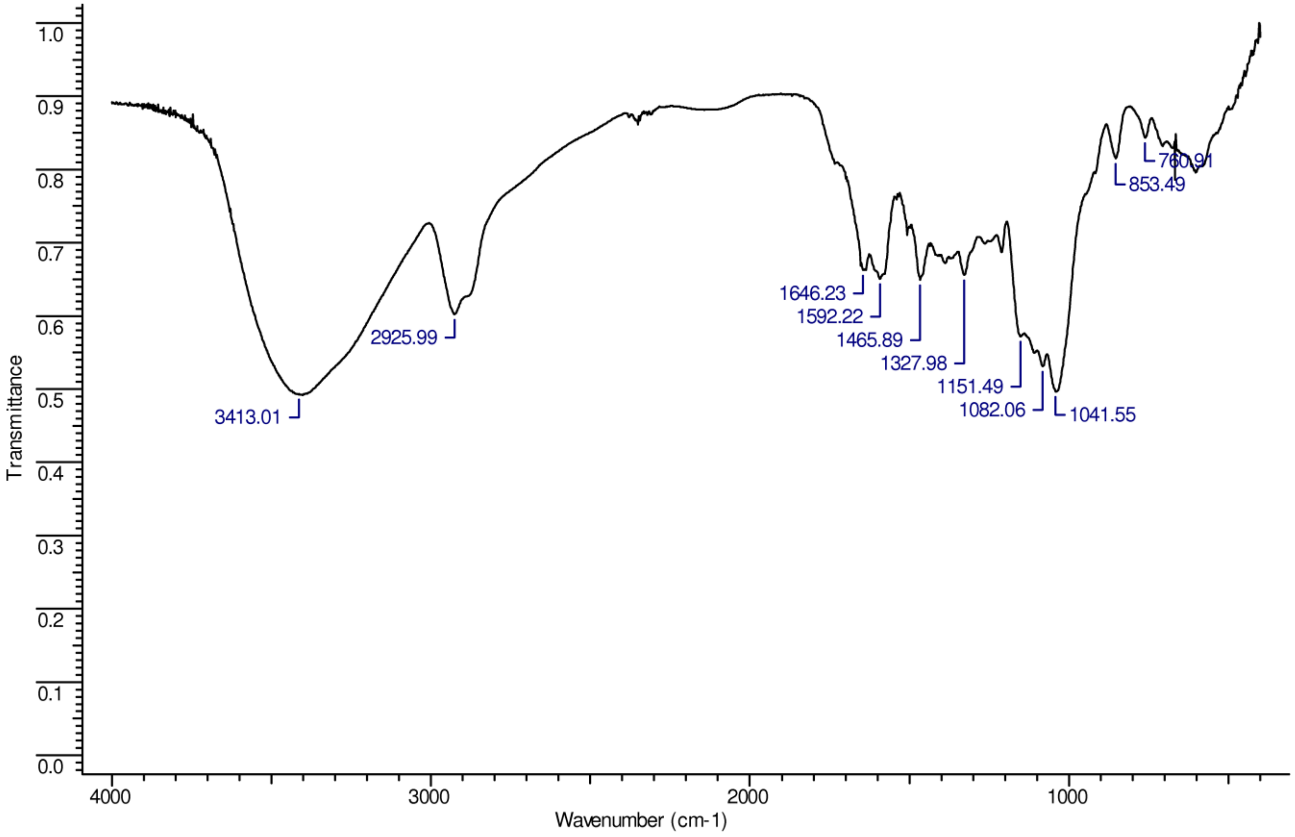

Figure S31

(6-amino-6-deoxy)$_n$-6-[(5/6)-fluoresceinylthioureido]$_m$- poly-β-CD **(10)**

^{1}H-NMR

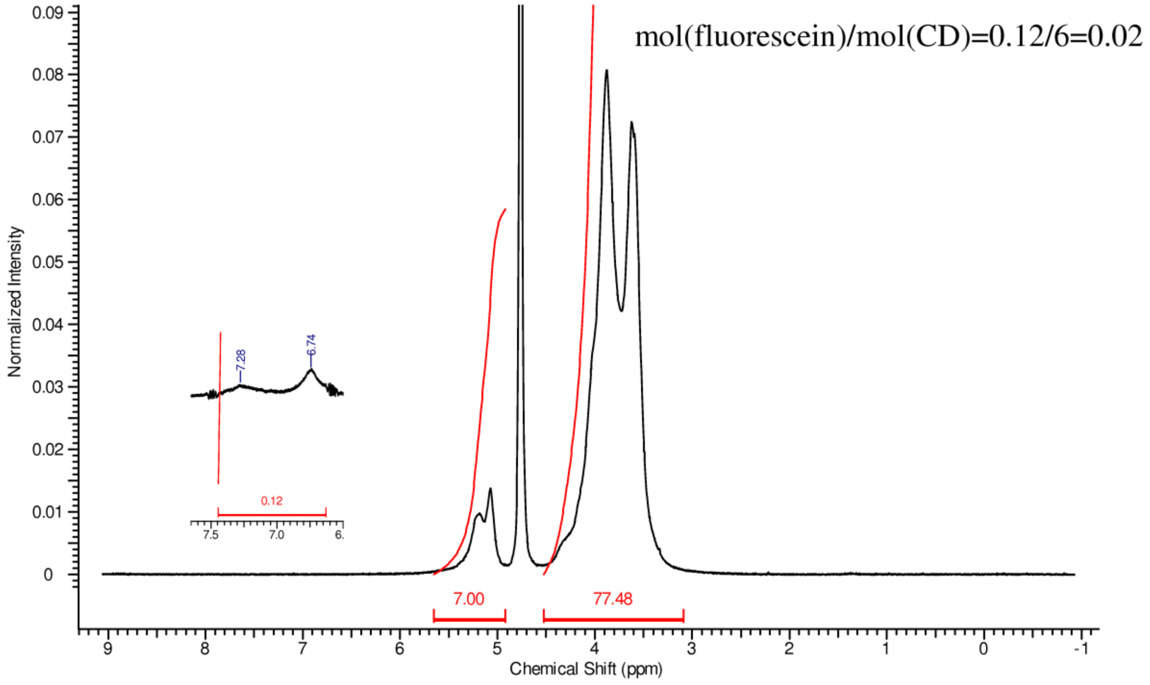

Figure S32

(6-amino-6-deoxy)$_n$-6-[(5/6)-fluoresceinylthioureido]$_m$- poly-β-CD **(10)**

HSQC-DEPT

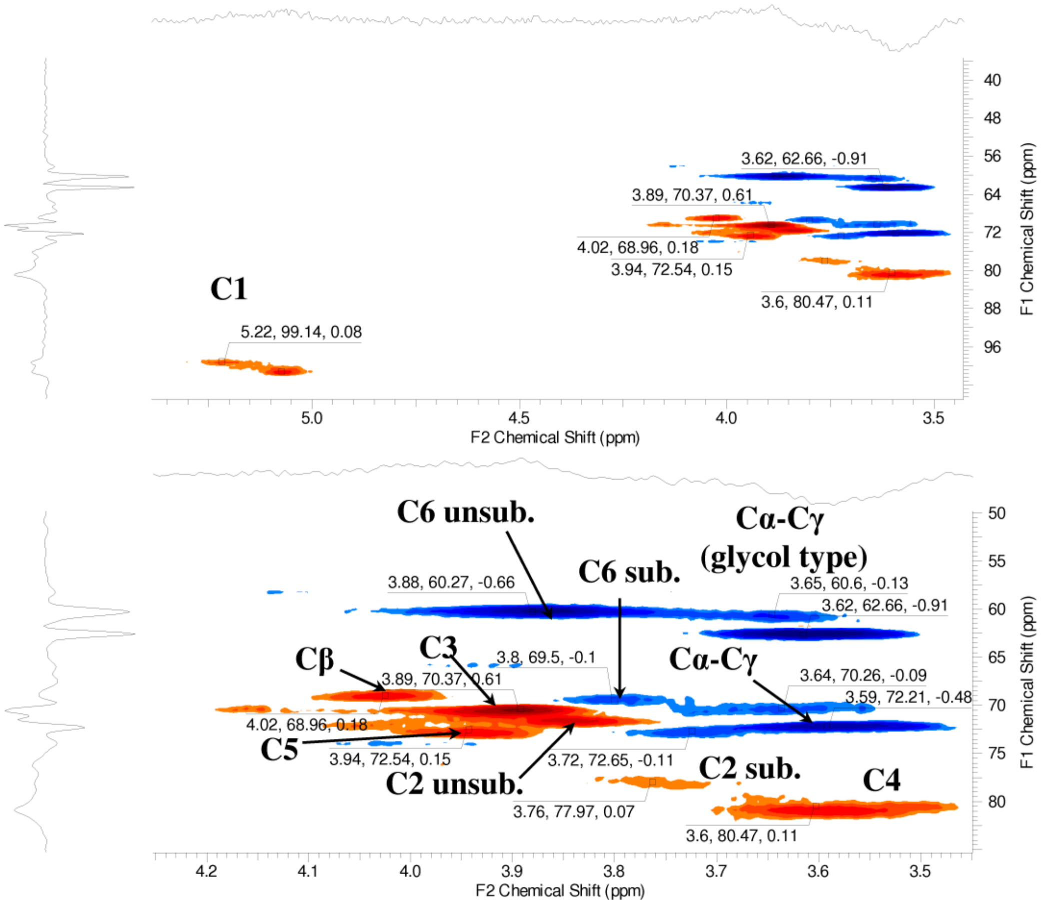

Figure S33
